# Data mining techniques in psychotherapy: applications for studying therapeutic alliance

**DOI:** 10.1038/s41598-023-43366-6

**Published:** 2023-09-29

**Authors:** Nasim Sadat Mosavi, Eugénia Ribeiro, Adriana Sampaio, Manuel Filipe Santos

**Affiliations:** 1 Algoritmi Research Centre, University of Minho, Guimarães, Portugal; 2 Psychotherapy and Psychopathology Research Lab, Centre for Research in Psychology (CIPsi), School of Psychology, University of Minho, Braga, Portugal; 3 Psychological Neuroscience Lab, Center for Research in Psychology (CIPsi), School of Psychology, University of Minho, Braga, Portugal

**Keywords:** Human behaviour, Computer science, Information technology

## Abstract

Therapeutic Alliance (TA) has been consistently reported as a robust predictor of therapy outcomes and is one of the most investigated therapy relational factors. Research on therapists' and clients’ contributions to the alliance development and the alliance-outcome relationship had shown mixed results. The relation of the therapist’s and client’s biological markers with the alliance is an important and under-investigated topic. Taking advantage of data mining techniques, this exploratory study aimed to investigate the role of different therapist and client factors, including heart rate (HR) and electrodermal activity (EDA), in relation to TA. Twenty-two dyads with 6 therapists and 22 clients participated in the study. The Working Alliance Inventory (WAI) was used to evaluate the client’s and therapist's perception of the alliance at the end of each session and through the therapy processes. The Cross-Industry Standard Process for Data Mining (CRISP-DM) was used to explore patterns that may contribute to TA. Machine Learning (ML) models have been employed to provide insights into the predictors and correlates of TA. Our results showed that Linear Regression (LR) was the best technique for predicting the therapist’s TA, with client “Diagnostic” and therapy “Termination” being identified as significant predictors of the therapist’s TA. In addition, for clients’ TA, the Random Forest (RF) was shown to have the best performance. The therapist’s TA and therapy “Outcome” were observed as the most influential predictors for the client’s TA. In addition, while the Heart Rate (therapist) was negatively associated with the therapist’s TA, EDA in the client was a physiological indicator related to the client’s TA. Overall, these findings can assist in identifying key factors that therapists should focus on to enhance the quality of therapeutic alliance. Results are discussed in terms of their consistency with empirical literature, innovative and interdisciplinary research on the therapeutic alliance field, and, in particular, the use of the Data Mining approach in a psychotherapy context.

## Introduction

Psychotherapy process research aims to study how psychotherapies work and which factors are associated with specific outcomes^1^. These objectives can be achieved by outcome and/or process-based research which include evaluating therapy outcome and/or critical aspects of the process of the in-therapy sessions as the behavior and the quality of interaction between both client and therapist, respectively^2^.

The therapist’s and client's mutual collaboration and agreement on the goals and task of the therapeutic work during the treatment process, along with a secure bond between the dyad, is usually referred to as therapeutic alliance^3,4^. This relational factor is considered effective and a robust predictor of therapy outcomes across different psychotherapy approaches and mental health diagnoses indicated by several meta-analyses^5–7^.

The alliance is then a dyadic and dynamic construct, to which the therapist and patient collaboratively contribute in the context of each session and across therapy sessions, despite different evaluations of TA across treatment may exist between the dyad^8^. Considering that a stronger alliance emerges from a negotiation process, it is understandable that the estimation of TA by both patient and therapist may diverge at the beginning of therapy^9^ although seems to converge over time and towards the end of successful therapies^6,10,11^. The success of this negotiation process benefits both patient and therapist by allowing them to form a trusting relationship and to engage in interpersonal and social processes such as affiliative and aligned interactions^12^. The available research on therapist variables mediating or moderating the quality of alliance has been focused on factors like interpersonal skills, communication skills, empathy, experience and training, and intrapersonal dimensions^13–15^. The study of the role of patients' contribution has pinpointed factors such as initial symptoms severity, type of diagnosis, patients’ interpersonal skills, or attachment style^8^. Furthermore, while some studies have looked at patient evaluation of alliance, others have considered both therapist and patient sides of the alliance, whether as independent or related perspectives^14,16^. Considering the subjective and dynamic nature of both therapist and patient evaluation of the quality of the TA, and the interpersonal process inherent to the alliance experience, there has been an increasing interest in analyzing other factors that may be related to the alliance development^6^ as biological variables (i.e., physiological reactivity)^17–19^. In accordance, some studies have suggested that physiological measures may capture other related characteristics (e.g., empathy; and engagement) that facilitate or hinder the formation of a working alliance and therefore can be useful objective measures for estimating the alliance. Evidence has shown a positive relationship between the client’s perception of therapist empathy and the therapist and client's physiological concordance, measured with skin conductance measures (EDA)^17^. The role of pre-therapy physiological variables has also been demonstrated, with a trauma-focused therapy study showing that lower pretherapy patients’ EDA predicted stronger working alliance at the end of 12 weeks of therapy^20^. These results are also consistent with intervention studies, i.e., with evidence showing variations in the client's physiological activity during therapeutic interactions being influenced by the therapist's behaviors^21,22^.

The analysis of heart rate (HR) is another physiological measure that has been studied in psychotherapeutic contexts^23^. A study on therapeutic alliance found that while both the therapist and client's heart rates decreased from the beginning of the therapy session towards the end, the therapeutic alliance increased^24^. Furthermore, a positive relation between in-session high-frequency heart rate variability (HRV) and the therapeutic alliance was documented in clients receiving cognitive behavior therapy^23^.

Physiological reactivity has been accepted as an interpersonal aspect that is essentially influenced by interactive exchanges occurring within a social context^25^. On the basis of the evidence currently available, the interplay between the two autonomic branches (sympathetic and para-sympathetic) seems to represent how easily individuals can shift their arousal states between high and low levels of reactivity. Therefore, physiological responses (i.e., through cardiac output and EDA), seem to be underlying social behaviors and emotional processes by influencing the perception of safety and, consequently, allowing engagement in social interactions. This is particularly evident in psychotherapy if we assume that the quality of the expressions of emotional and cognitive processes, which occur throughout the therapy process and between the therapeutic dyad, influence the way both therapist and client join in therapeutic interactions, and consequently construct the therapeutic alliance. However, until now, studies focused on the relationship between the quality of therapeutic alliance and therapist and client in-session physiological activity are limited to the understanding of the role of the parasympathetic nervous system (e.g., HRV) in the therapeutic alliance^23,24^ or based on the analysis of interpersonal synchrony patterns^26^. Therefore, in this study, we have considered the therapist and patient physiological data, using both EDA and Heart Rate, as possible barometers of the therapeutic alliance.

Integrating clinical and physiological data in-session and throughout sessions for both clients and therapists require highly complex analytical models. While the traditional statistical approaches allow us to analyze the relationship between measures, their power to provide important information on the specific temporal nature of the data, particularly physiological data, and the complex relation between different psychotherapy measures is limited^27^. Recent advancements in the field of computer science resulted in a strong development of predictive models using large amounts of data with Data Mining (DM) and Machine learning (ML) techniques that proved to be relevant for mental health care^28^ and specifically for psychotherapy^29,30^. These techniques have been proposed as a promising tool for addressing the complexity of the psychotherapy process, namely by accounting for the dynamic process that occurs between therapist and client throughout the therapy^29,31,32^. A scoping review exploring broadly the applications of ML in psychotherapy^29^ identified fifty-one studies, from which 44 were aimed to develop or test ML models and to inform on methods and applications of ML in the context of psychotherapy. The authors have concluded the current applications of ML to the treatment process, adherence, therapist skills, and treatment response prediction, as well as ways to accelerate research through automated behavioral or linguistic process coding. Specifically in the context of therapeutic relationship research, some studies have demonstrated the relevant application of ML to assess therapists' interpersonal and relational skills^33^. Specifically, some studies focusing on therapeutic alliance^31^ have discussed the applicability of machine learning and natural language processing to session recordings to predict the client-rated therapeutic alliance by using a large naturalistic psychotherapy dataset. Based on their results, the authors concluded that linguistic signals were indicative of the strength of the alliance, showing that ML techniques can be a useful tool for analyzing therapeutic alliances. In this line, Zhou et al.,^34^ also employed deep learning algorithms to predict first session alliance successfully.

ML techniques may contribute to the understanding of the role of TA as an in-session emerging and dynamic interpersonal process, by analyzing the underlying neurophysiological substrate at a dyadic level (client and therapist). Although biological variables such as HR and EDA are more diffuse constructs (not reliably observable) than others analyzed in ML studies in psychotherapy^31^, they might be reliable signals of in-session dynamics as they are related to empathy, safety, engagement, compassion, and emotional co-regulation, i.e., variables associated with the therapeutic alliance^35,36^. Therefore the objective of this exploratory study was to leverage data mining techniques to analyse and uncover meaningful patterns in a psychotherapy dataset including therapeutic alliance data. We aimed to identify significant factors or variables that influence the strength of the therapeutic alliance and gain insights into the dynamics and characteristics that impact this vital factor of the therapeutic process. By employing data mining techniques, the study seeks to unveil hidden patterns, relationships, and trends within the data, thereby advancing our comprehension of therapeutic alliance and potentially guiding therapeutic practices, by addressing two exploratory questions: How can data mining/ML techniques be applied to explore and gain insight into the therapy factors that influence therapeutic alliance to effectively predict therapeutic alliance using diverse sets of data of clients and therapists, encompassing both physiological and psychological factors?

## Method

### Participants

The sample consisted of 18 women and 8 men, with ages ranging between 18 and 52 years (*M*_*age*_ = 29.1, *SD* = 11.08). From this initial sample, three did not initiate the psychological treatment (abandoned the project after the screening interview) and one client did not have a physiological recording (ID1). Therefore, the final sample included twenty-two clients (fifteen clients were diagnosed with Major Depressive Disorder, and seven clients were diagnosed with Social Anxiety Disorder). The diagnoses were performed using the Structured Clinical Interview for DSM-IV-R Axis I Disorders^37^. Exclusion criteria considered Axis II diagnosis or any other concurrent Axis I disorder that could be the focus of clinical attention, such as suicidal ideation, or psychotic symptoms. Therapists who participated in the study included 5 women and 1 man with clinical experience ranging between 4 and 22 years (*M*_*years practice*_ = 10.3, *SD* = 6.25), all with cognitive behavior training (CBT). The CBT protocol included 16 weekly sessions (around 60 min per session) and two monthly follow-up sessions.

### Instruments

The therapeutic alliance was measured with the Portuguese version of the Working Alliance Inventory-Short Revised (WAI-SR)^38^. WAI-SR assesses the therapeutic alliance’s quality based on a scoring system that yields three dimensions: (1) agreement on tasks; (2) agreement on goals, and (3) development of a bond. The total score of WAI-SR depends on each of the three scores. The WAI-SR (client) includes 12 items rated on a 5-point (Likert scale) ranging from 1 (seldom) to 5 (always). Higher scores in this measure reflect a better-working alliance and total scores ranged from 12 to 60. Similarly, the therapist’s WAI-SR includes 10 items rated on a 5-point (Likert scale), ranging from 1(seldom) to 5 (always), and total scores ranking, ranging from 10 to 50. The Portuguese version of WAI-SR has good psychometric properties concerning its items' sensitivity, factorial structure, and fidelity^38^. A study of the fidelity of the Portuguese version of WAI-SR, reveals Cronbach's Alpha values (0.85 for the global scale), which are under values reported in the literature^39^. In the current study, the therapeutic alliance was independently evaluated from both the therapist's and the client’s perspectives, at the end of each therapy session. We obtained both Cronbach’s Alpha and McDonald’s Omega values for the WAI-SR client [global scale—Cronbach’s Alpha of.93 and McDonald’s Omega total of 0.95) and for the WAI-SR therapist [global scale—Cronbach’s Alpha of 0.88 and McDonald’s Omega total of 0.91).

Physiological data. The BioNomadix system (BIOPAC Systems, Santa Barbara, CA, USA) was used to collect the cardiac activity and electrodermal activity, with a sampling rate of 1000 Hz. The cardiac data was acquired through the BN-RSPEC module connected to the BioNomadix, using a sampling rate of 1000 Hz. The cardiac activity was recorded as Heart Rate (HR), measured in beats per minute, achieved synchronously from both participants through an Electrocardiogram (ECG). The electrode placement was based on an adjusted 3-electrode Lead-II configuration, placed on the participants’ left middle of the clavicle and a third one on the left spine of the scapula. The HR electrodes were filled with an electrode gel intended explicitly for recording bioelectrical potentials. The disposable Ag–AgCl electrodes (Type EL-503, BIOPAC Systems Inc.) were attached after the skin cleaning procedure with alcohol and dried with cotton to diminish impedance and improve signal quality. The raw ECG data were filtered using the recommended standard filter settings for the acquisition device, an IIR high-pass filter of 1 Hz and an IIR low-pass filter set at 35 Hz.

The data acquisition system for electro-dermal activity was directly coupled to two electro-dermal amplifiers (BN-PPGED module for the SCL), one for each participant in the dyad (i.e., one for the client and another one for the therapist), allowing the synchronous and simultaneous recording of the data. This BioNomadix system was also connected to a computer running the Acknowledge 4.4 software (BIOPAC Systems, USA), which allowed the acquisition and storage of all the physiological data in real time. The electro-dermal amplifier, placed on the participants’ non-dominant wrist, was connected to disposable silver/silver chloride electrodes (Type EL-507, BIOPAC Systems Inc.) placed on the palmar surface of the medial phalanges of the index and middle fingers of the non-dominant hand, on the therapist and the client. Before electrode placement, all participants were asked to wash their hands with water and a non-abrasive soap to ensure a close degree of skin hydration. To measure the skin conductance level (SCL) of the electrodermal activity, the transmitter BN-PPGED passes a constant voltage of 0.5v between the two sensors and transfers the difference in charge (i.e., the conductance afforded by the sweat glands on the palm) back to the BioNomadix data acquisition unit. Then, after the data collection, the raw SCL signal was visually inspected and then filtered using the recommended standard filter settings for the acquisition device, a FIR low-pass Blackman filter of 1 Hz with the number of coefficients set at 4000^40^.

The HR was calculated offline from the filtered ECG trace and the SCL was analyzed using the Acknowledge 4.4 software with 1-min epochs being calculated for each participant in all sessions. Therapists' and clients’ physiological activity was recorded simultaneously throughout each session and across all therapy sessions.

### Procedure

The dataset for the current study is part of a major research project focused on therapeutic collaboration and its physiological correlates (grant BIAL-178/12). This study was approved by the Ethical Committee from the Research Centre in Psychology of the University of Minho (November 16, 2012). Moreover, all methods were carried out following guidelines for research in psychology, enshrined in the Declaration of Helsinki of the World Medical Association (WMA) (2008). Informed consent was obtained after explaining the procedure to the participants in a prior meeting at the beginning of the treatment. Both therapists and clients accepted all the procedures involved in the data collection, were permitted to use their data, and signed the informed consent in writing form. The therapeutic process was offered free of charge, and it was assured that, if the client wanted to withdraw his participation from the research project, the therapy process would continue, if necessary.

At intake (session 0), the therapist conducted an initial and structured evaluation to verify the project’s inclusion criteria. Furthermore, psychiatric and/or physical comorbidities were assessed to exclude the possibility of confounding the results concerning the physiological measures. The therapist and the client were both asked to refrain from caffeine, physical exercise, and nicotine for at least 4 h before each session. At the time of the sessions in analysis, no one was taking any medication. For the purpose of assessing the baseline level of the client and the therapist’s physiological measures, a 10-min minimally demanding baseline task was performed by both the client and the therapist before the beginning of each therapeutic session. The therapist instructed the client that some images would be presented to them in the centre of a laptop screen and, together, they should describe as many details as possible for each image. The stimuli were coloured and neutral objects on a white background. In an adjacent room, the computer for recording the physiological activity was placed, and a team was responsible for following the recording during the baseline and therapy session. Manual markers along the register were inserted to synchronize the different therapeutic session moments (baseline task, therapeutic session) and physiological activity. The therapist was responsible for providing a signal that would represent the beginning of each therapeutic moment. Furthermore, the therapist and the client were invited to report any discomfort or subjective effects related to the psychophysiological recording devices.

The Cognitive Behavior Therapy protocol included 16 weekly sessions (around 60 min per session) and two monthly follow-up sessions. After each therapy session, therapist and client independently filled in the respective WAI form, and introduced it in an envelope to be picked up by a research team member.

### Process

For the current study, we have used CRISP-DM 1.0. approach. CRISP-DM stands for CRoss-Industry Standard Process for Data Mining. Data Mining (DM) refers to the process of applying intelligent techniques to data to extract patterns and identify valid and useful information^41^.

**Figure 1:**
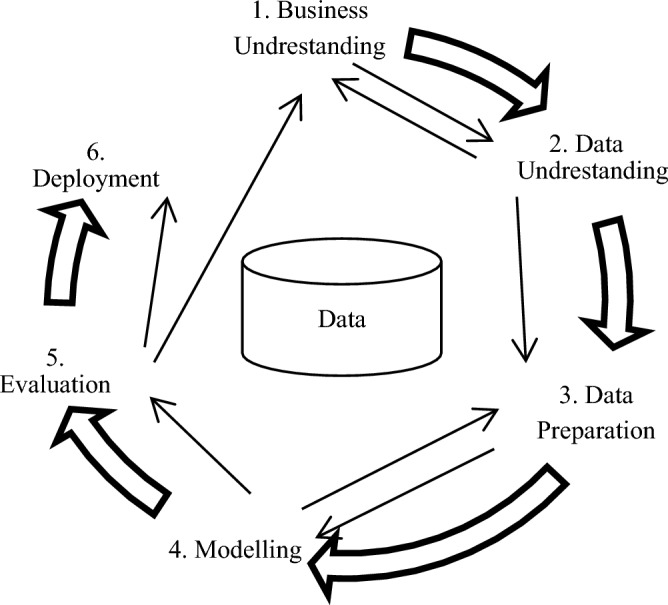
CRISP-DM process.

Whereas Data Mining (DM) is one of the phases in the Knowledge Discovery from Database (KDD) process for searching and discovering patterns, the CRISP-DM guides people to know how DM can be applied in practice in real systems^42^. CRISP-DM is a standard methodology used to support translating business problems or application requirements and objectives into data mining projects. Regardless of the type of industry, CRISP-DM helps the effectiveness of the outcome by extracting knowledge from the raw data^42^. This methodology was introduced in the late 90 s for Knowledge Discovery from Database (KDD) and was developed by a consortium initially composed of Daimler-Chrysler, SPSS, and NCR. As Fig. 1 shows, CRISP-DM 1.0. includes six phases and each phase includes tasks and outcomes. (1) Business/Application Understanding (identify the business /data mining objectives and goals,determine how data mining can help achieve those objectives) (2) Data Understanding (explore the available data via data analysis tasks, assess the quality and suitability of the data, identify data issues and potential challenges), (3) Data Preparation (select and preprocess the relevant data, cleanse, transform, and integrate the data as required. and perform feature engineering and selection), (4) Modeling (select appropriate modeling techniques, build and train the models using the prepared data, and evaluate and refine the models for performance improvement), (5) Evaluation (assess the model's effectiveness in achieving the business objectives, validate the model's performance, and identify areas for improvement and fine-tuning), and (6) Deployment (integrate the model into the business operations or decision-making process, create a plan for model deployment and monitoring and document the final results and provide recommendations)^42,43^.

### Tools and techniques

In this experiment, we have employed six regression algorithms including Artificial Neural Network (ANN), Random Forest (RF), Decision Tree (DT), K Nearest Neighbors (KNN), Support Vector Regression (SVR), and Linear Regression (LR). Moreover, we have accounted for nested cross-validation to rank algorithms. In addition, regression metrics such as Coefficient Determination (R2) and Mean Squared Error (MSE) assessed the performance of each algorithm.

In terms of tools, we have used Microsoft Power BI to figure out, visualize the critical relationship, and identify possible patterns in the data-understanding phase. Furthermore, the data analysis, modeling, and evaluation were performed using the Python libraries such as Pandas and Sikit-learn.

## Results

This section presents the main results according to the different CRISP-DM phases and tasks to answer the research question of how can data mining/ML techniques be applied to explore and gain insight into the therapy factors that influence therapeutic alliance.

### Application/business understanding

In this phase of the CRISP-DM process, a crucial task was to define both business and data mining objectives separately. The aim was to uncover how data mining can add value to business applications, and align the goals of the data mining approach with the overall business objectives.

Therefore, in this line, we sought to identify the key factors or variables that significantly impact the strength of the therapeutic alliance between clients and psychotherapists. Additionally, we explored the impact of the dynamics and characteristics of the therapeutic process on the therapeutic alliance.

As part of the data mining goals, the study aimed to uncover hidden patterns, relationships, and trends within the dataset. This exploration was crucial for enhancing our understanding of therapeutic alliance. Moreover, the study intended to evaluate the accuracy of machine learning models in predicting the level of therapeutic alliance based on several client and therapist factors, sessions, and therapy variables. Another objective was to determine which machine learning algorithms outperform others in predicting therapeutic alliance. Based on that, the study aimed to provide insights into the most effective approaches for predicting therapeutic alliance by identifying the top-performing algorithms.

Considering the business goals, the study intended to utilize the findings to develop data-driven approaches for assessing and optimizing therapeutic alliance. Given the well-documented relationship between therapeutic alliance and therapy outcome, we sought to explore how the application of data mining and machine learning can contribute to improving treatment outcomes and potentially support clinical decision-making in psychotherapy.

Based on that, we have analyzed the scores of the Portuguese version of WAI-SR. Hence, this study considered the total WAI-SR (TA) score for both clients and therapists (WAI-SR-Client, WAI-SR-Therapist). In other words, the target for prediction was defined as the total TA.

### Data understanding

Based on the data analysis, the initial dataset included 24,525 rows and 45 variables with 13,6767 missing cells. Table 1 shows the description of each variable. The client’s ID and therapist’s ID are the unique identifications of clients and therapists. There are six therapist_ID and twenty-two clients_ID. Each client was assigned to one therapist for all therapy sessions, but each therapist could attend to different clients. “Sex” was classified as male (sex = 0) or female (sex = 1). Moreover, the “Diagnosis” shows if the record is associated with anxiety (diagnostic = 0) or depression (diagnostic = 1), referring to the client's diagnosis. The “Outcome” identifies the result of each therapy process, as poor (outcome = 0) or good (outcome = 1). In addition, the “Termination” shows if the client is a dropout (termination = 0) or completed (termination = 1). The number of therapy sessions is displayed as “Session”, with each session being fragmented in epochs with a fixed period of one minute. The “Time” shows the period in seconds, almost equal to sixty epochs (in each epoch/session number). The value of “Condition” is constant as “1” showing the under-treatment status and “0” the baseline before the session. Moreover, biological variables for both clients and therapists included Heart Rate (mean, mean_baseline, SD_baseline, standardized) and EDA (mean, mean_baseline, SD_baseline, standardized). The value of EDA and HR reported in the context of _baseline, SD baseline, and standardized are repeated values for the different epochs at the same session, at baseline. However, the HR (mean) and EDA (mean) values are different in each epoch, as they refer to the in-session period. Finally, the WAI-total score which will be discussed as “WAI” in technical machine learning represents the value of TA for the client and therapist (client’s WAI, and therapist’s WAI, respectively).

**Table 1:** Description of variables*.*

**#**	Name of the variables	Description
1	ID	Identification number; (client and therapist)
2	Sex	man = 0; = 1woman;(client and therapist)
3	Diagnostic	0 = anxiety; 1 = depression
4	Outcome	0 = poor; 1 = good
5	Termination	0 = dropout; 1 = completed
6	Session	Number of the therapy session
7	Condition	baseline = 0; treatment = 1
8	Epoch	The time interval of 1 min
9	Time	Spent time in the therapy session
10	HR (Mean)	Heart Rate-mean (bpm); (client and therapist)
11	HR (Mean-Baseline)	Heart Rate- baseline (bpm); (client and therapist)
12	HR (SD-Baseline)	Heart Rate-standard deviation (bpm); (client and therapist)
13	HR (Standardized)	Heart Rate- standard (bpm); (client and therapist)
14	EDA (Mean)	Electrodermal Activity – mean; (client and therapist)
15	EDA (Mean Baseline)	Electrodermal Activity. Baseline; (client and therapist)
16	EDA (Standardized)	Electrodermal Activity-standard; (client and therapist)
17	EDA(SD-Baseline)	Electrodermal Activity- standard deviation; (client and therapist)
18	WAI score (TA)	The total value of TA; (client and therapist)

Table 2 shows descriptive statistics including those that summarize the central tendency, dispersion, and shape of a dataset’s distribution (excluding missing values). The statistic includes count (none missing values), mean, standard deviation (sd), minimum (min), and maximum (max) for selected variables (epoch, time, TA, EDA (mean), HR (mean). The number of records, excluding missing values, is equal to 23079. The minimum epoch was 1 and the maximum was 111. In addition, the minimum number of therapy sessions was 1 and the maximum was 18, with the mean value of the total session being equal to 8.52.

**Table 2:** Descriptive statistics.

Epoch session time (s)				Client			Therapist		
				TA	EDA (mean)	HR (mean)	TA	EDA (mean)	HR (mean)
Count	23,079	23,079	23,079	23,079	23,079	23,079	23,079	23,079	23,079
Mean	35.96	8.52	2162.36	51.69	5.17	86.69	44	4.22	83.75
sd	22.62	4.95	1356.80	7.28	3.09	12.09	4.24	2.28	13.58
Min	1	1	00.00	27	− 4.27	00.00	27	-4.61	00.00
Max	111	18	7298	60	19.47	119.96	50	16.78	157.12

In terms of the duration of therapy sessions (time), the average time spent for therapy sessions was 2162.36 minutes (sd 1356.80 min). In addition, the minimum and maximum time were equal to 00.00 and 7298 min.

This analysis shows the mean value of the client’s TA was 51.62 (sd equal to 7.28), and the minimum and maximum were equal to 27 and 60, respectively. The same analysis for the therapist’s TA presents a mean value of 44 with sd equal to 4.24, a minimum therapist’s TA of 27, and a maximum value equal to 50.

Regarding the distribution of HR’s value and EDA’s value, the average of the client’s HR (mean) is 86.69 with the sd equal to 12.09, the minimum HR (mean) was 00.00 the maximum was 119.96. HR for therapists was 83.75 with the sd equal to 13.58, with minimum and maximum values of therapist’s HR ranging between 00.00 and 157.12. Similarly, in regards to EDA, the average value of clients’ EDA (mean) was 5.17 and this value for therapists was 4.22. Furthermore, the minimum and maximum values of the client’s EDA were observed as (−) 4.27 and 19.47. These values for therapists were (−) 4.61 and 16.78.

Developing effective prediction models strongly depends on the knowledge created via the modeling phase. Therefore, the understanding of the relationship among variables and finding patterns from the dataset is a useful input for modeling, which applies to business actions and decision-making. Thus, in this phase of Data Understanding, we have used Microsoft Power BI software to observe and analyze the possibility of three major relationships: TA by session, gender, and by ID (specific therapist and client). In addition, we have analyzed the number of therapy sessions attended by clients. Finally, we have investigated the possibility of existing linear relationships between HR and EDA with TA.

### Therapeutic alliance by session—client

**Figure 2:**
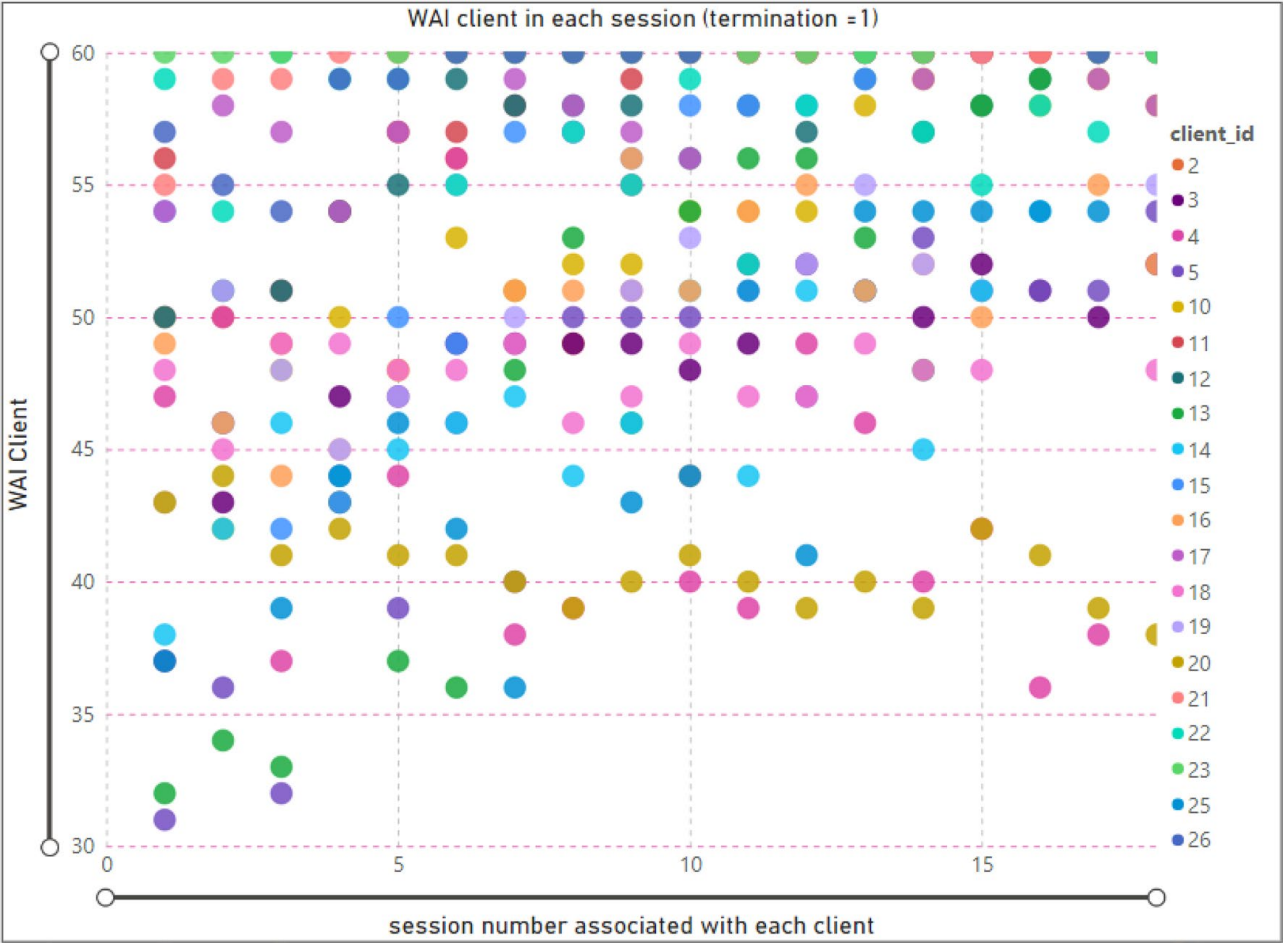
Relationship between session and WAI score (TA) for clients; termination = 1(completed).

In Figure 2, we display the relationship between the number of therapy sessions and TA for clients who completed the therapy (when the termination is equal to one). Each data point of the scatter chart represents the client ID (legend), session number (x-axis), and the value of TA for that specific session (y-axis). For example, the selected data point presents information about client ID5, in session 3, and a TA value of 32.

According to the scatter chart, some clients in the initial sessions presented a high value of TA, and others with more sessions showed less value of TA. For instance, the value of TA of client ID = 20, in session number 2 is 44, and in session 18 was 38. Moreover, the TA associated with client ID = 23 showed a maximum value (60) in all therapy sessions, and the client ID =13 showed an increased WAI with an increasing number of sessions.

**Figure 3:**
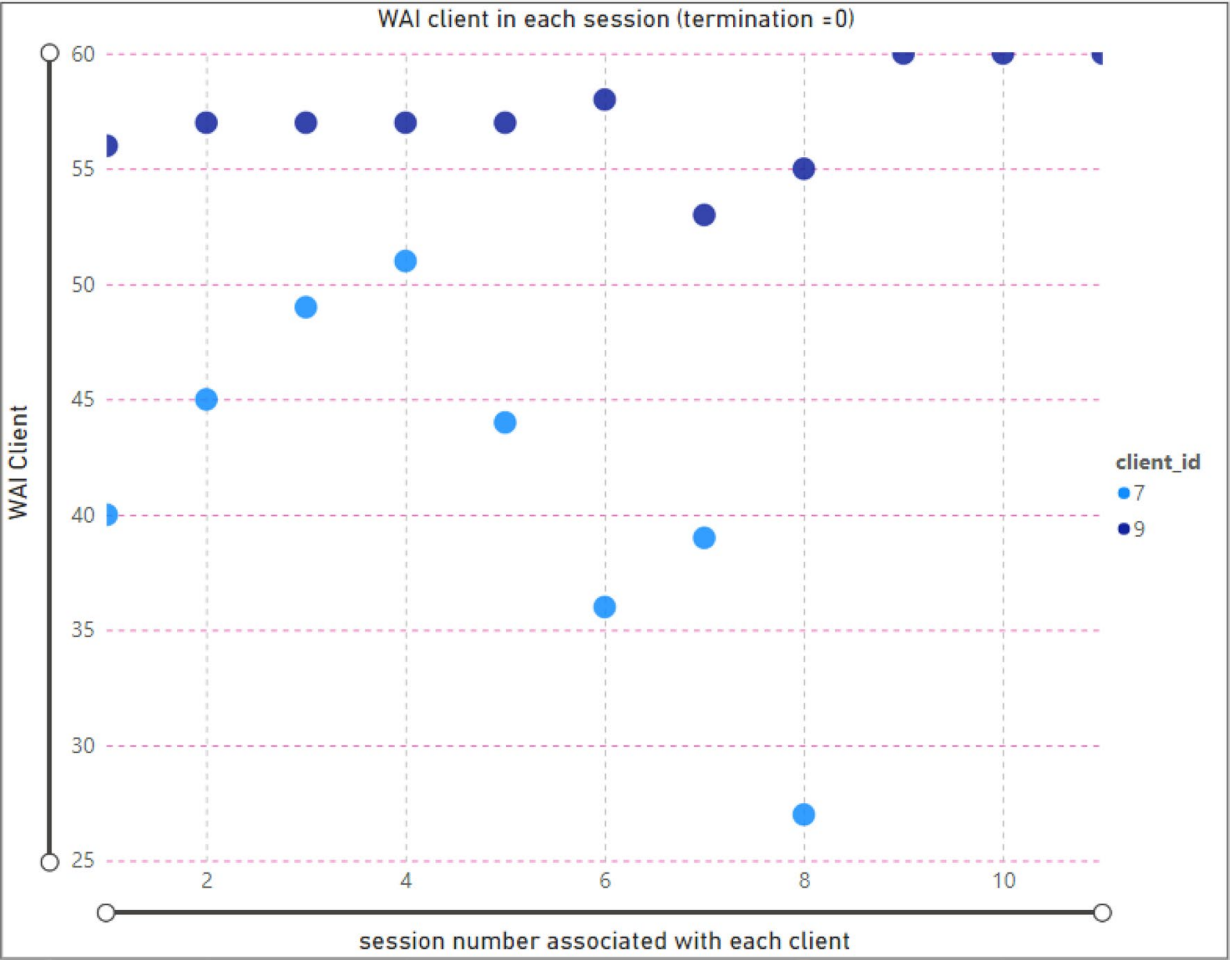
Relationship between session and WAI score (TA) for clients; termination = 0 (dropout).

Figure 3 shows the same analysis for clients who dropped therapy (where the termination is equal to zero). According to the scatter chart, there are only two clients who dropped therapy, one with 8 sessions (ID 7) and the other with 11 sessions (ID 9). The example shows the TA value of client ID 7, which is equal to 27 (in session 8).

### Therapeutic alliance by session—therapist

**Figure 4:**
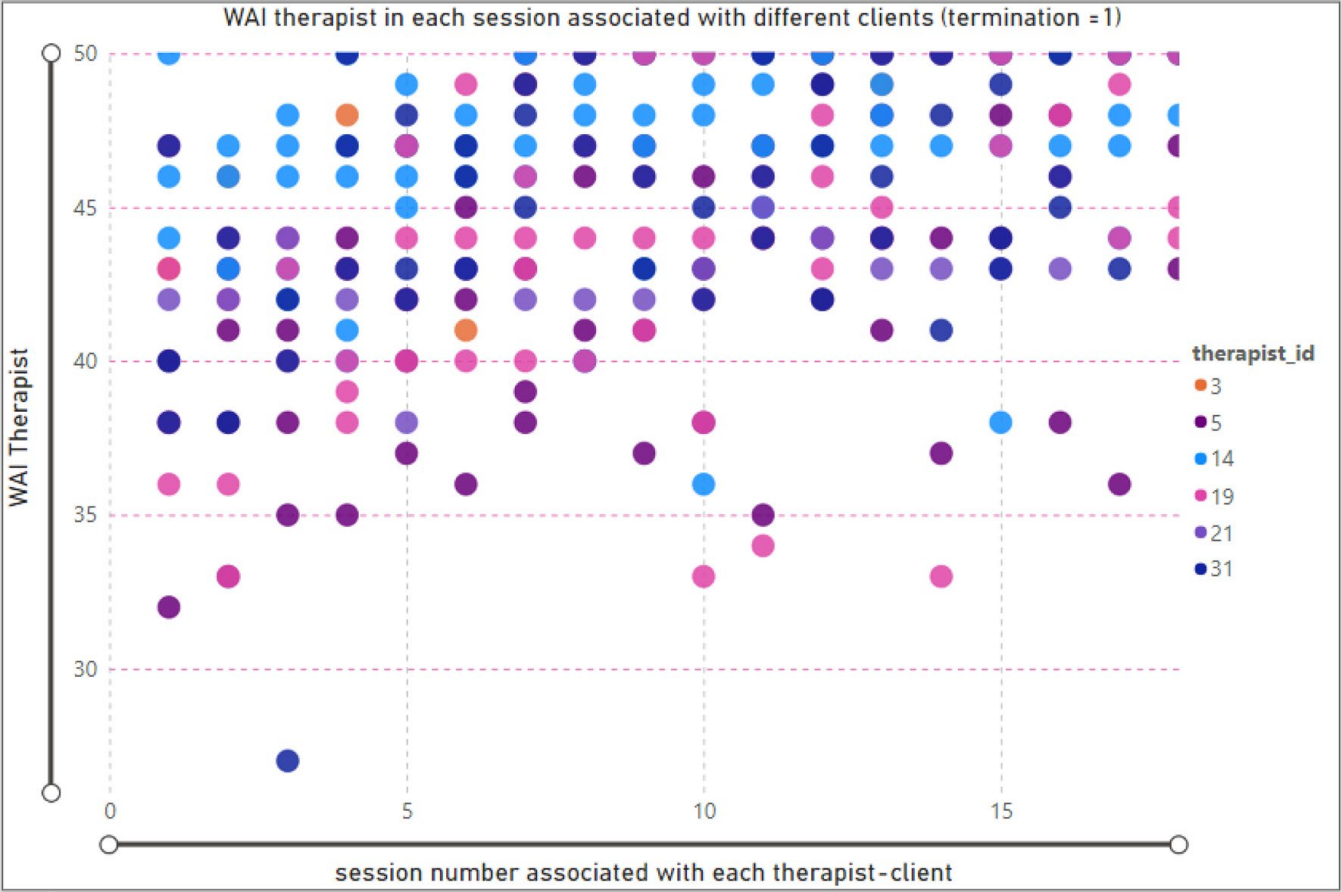
Relationship between session and WAI score (TA) for therapists; termination = 1(complete clients).

To analyze the relationship between the therapist’s TA and the number of therapy sessions, we have considered the client ID, since each therapist might have more than one client. Figure 4 presents the scatter chart displaying the therapist ‘ID, and the number of clients, in each session. The selected data points show that the minimum TA (27) was associated with therapist ID = 31 in session number = 3.

**Figure 5:**
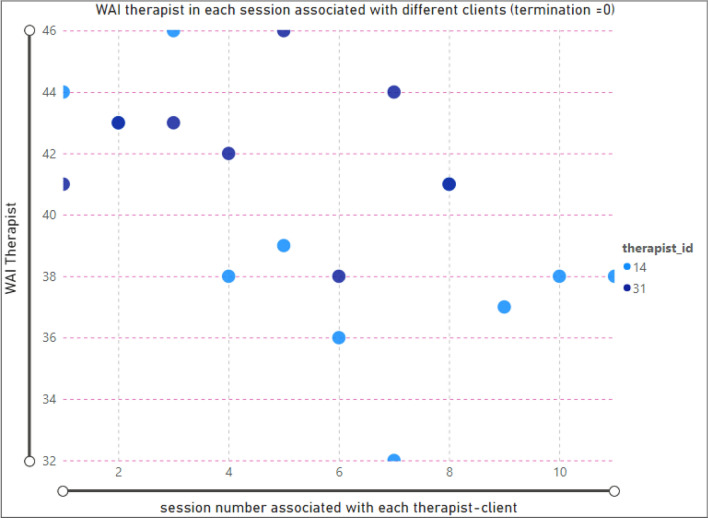
Relationship between session and WAI score (TA) for therapists; termination = 0 (dropout clients).

According to Fig. 5, there are two therapists (ID = 14, 31) that have sessions with dropout clients. The selected data points show that the TA value for therapist ID = 31 in session one is equal to 41.

### Therapeutic alliance by sex—client

**Figure 6:**
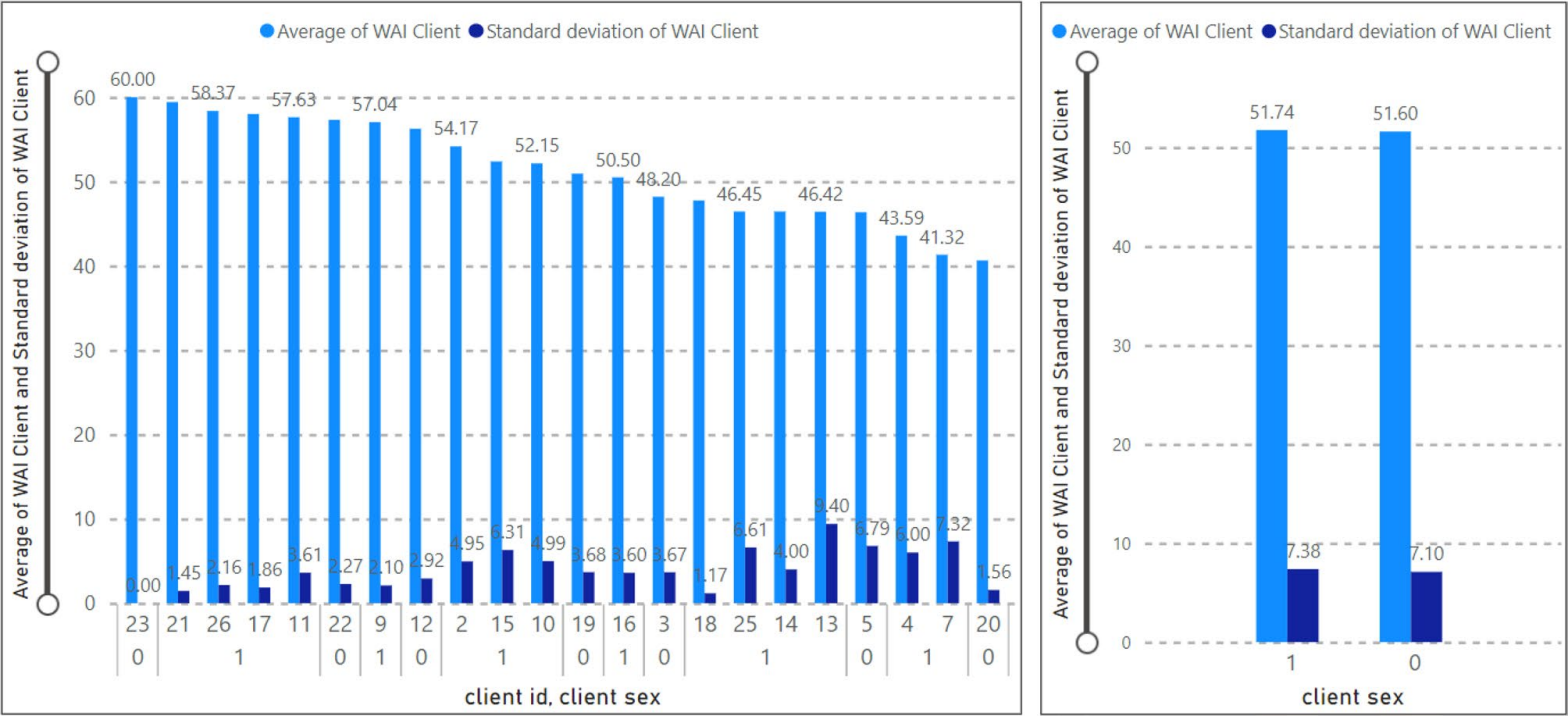
Average of WAI score (TA) by sex—client.

Figure 6 demonstrates the average value and standard deviation of TA given by the client’s sex (“client_sex” is equal to zero if male; or if it is equal to one, the client is female). Based on this analysis, out of twenty-two clients, seven of them were male (client ID = 3, 5, 12, 19, 20, 22, 23) and the remaining were female (client ID = 2, 4, 7, 9 , 10, 11, 13, 14, 15, 16, 17, 18, 21, 25, 26). In Fig. 5, the mean value of female clients’ TA was 51.74, and male client’s TA was 51.60. Moreover, the standard deviation of TA for female clients is equal to 7.38 and this value for male clients is equal to 7.10.

### Therapeutic alliance by sex-therapist

**Figure 7:**
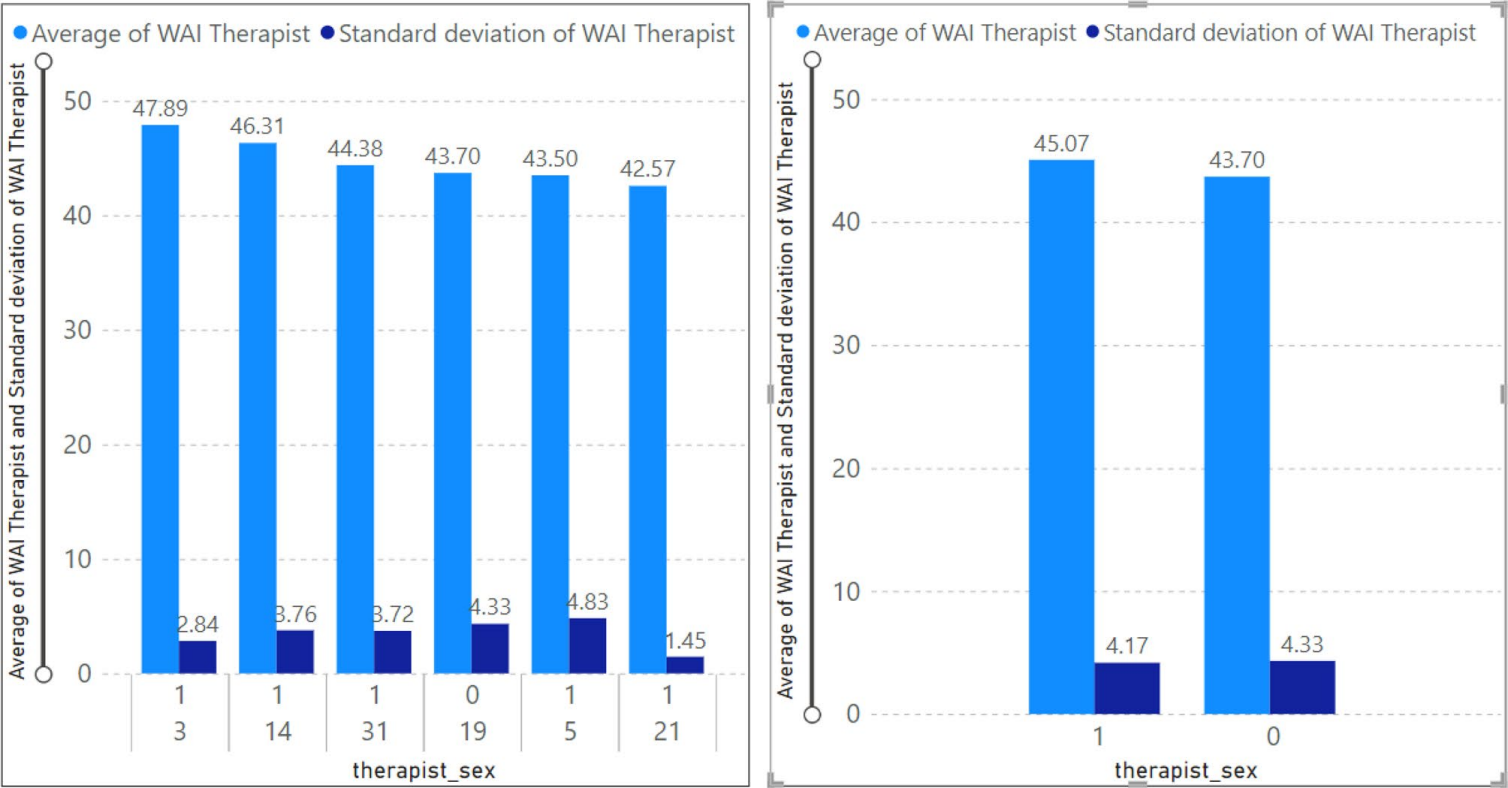
Average WAI score (TA) by sex-therapist.

Based on the TA by sex analysis (Fig. 7), we can observe that only therapist ID= 19 is male (therapist sex = 0), and the average TA value associated with this therapist is 43.70. Furthermore, the average value of TA belonging to other female therapists (therapist id = 3, 5,1 4, 21, 31) is equal to 45.07. Thus, female therapists showed a higher TA value than male therapists. According to Fig. 6, the TA’s sd for therapist ID equal to 19 is 4.33, and for female therapists is 4.17.

### Therapeutic alliance for specific clients

**Figure 8:**
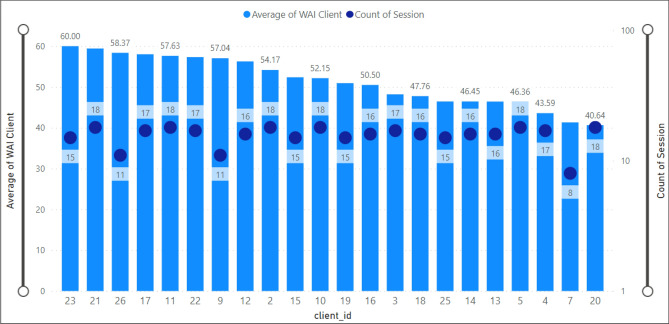
Average client’s WAI score (TA) by client id.

**Figure 9:**
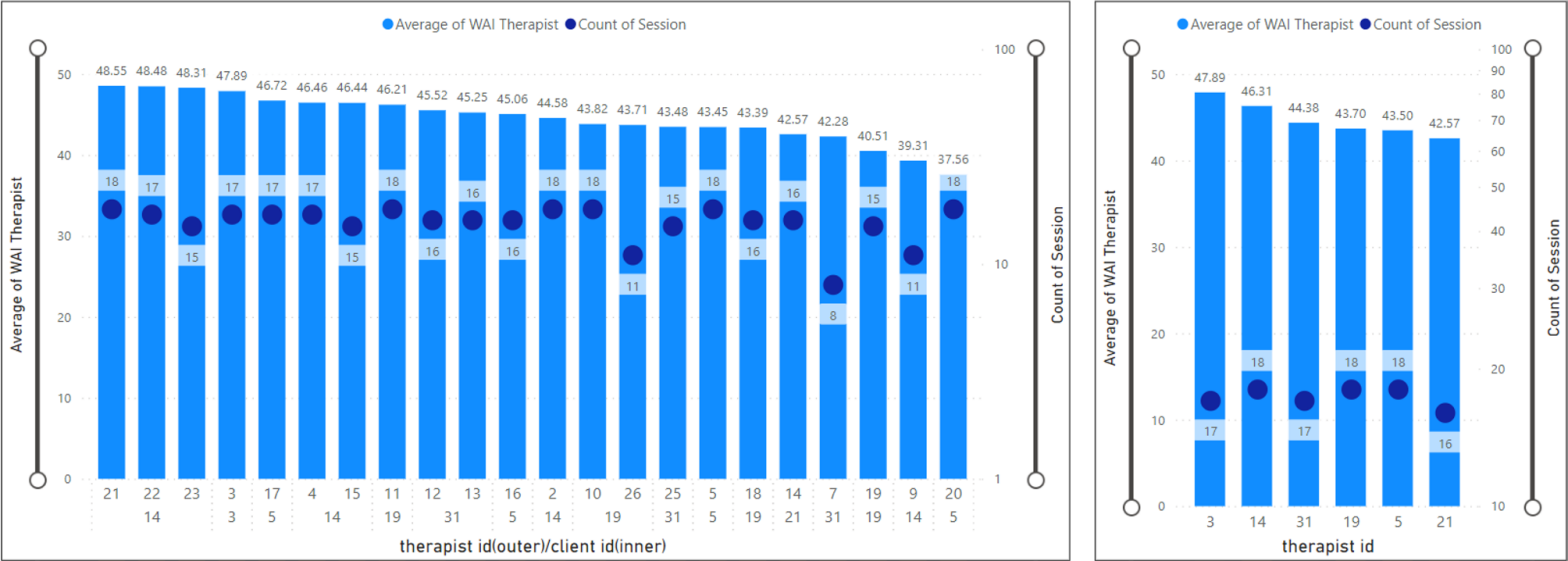
Average therapist’s WAI score (TA) by therapist ID.

In this analysis (Fig. 8), we have presented the average scores registered by IDs. Figure 9 illustrates that the minimum average value of TA was 40.64, registered by the client with ID number 20, whereas the maximum score was 60.00, given by the client with ID number 23.

### Therapeutic alliance for a specific therapist

Similarly, therapist ID number 3 had the maximum average value of WAI score (i.e., 47.89) and the minimum average was recorded by the therapist with ID number 21 (42.57). Considering that each therapist has more than one client, Fig. 9 shows the therapist's ID associated with a specific client. Specifically, therapist ID 14 with client ID 21 has an average TA value of 48.55 and the least average value of the therapist’ TA belongs to therapist ID 5 with client ID 20 (37.56).

### Sessions for client

**Figure 10:**
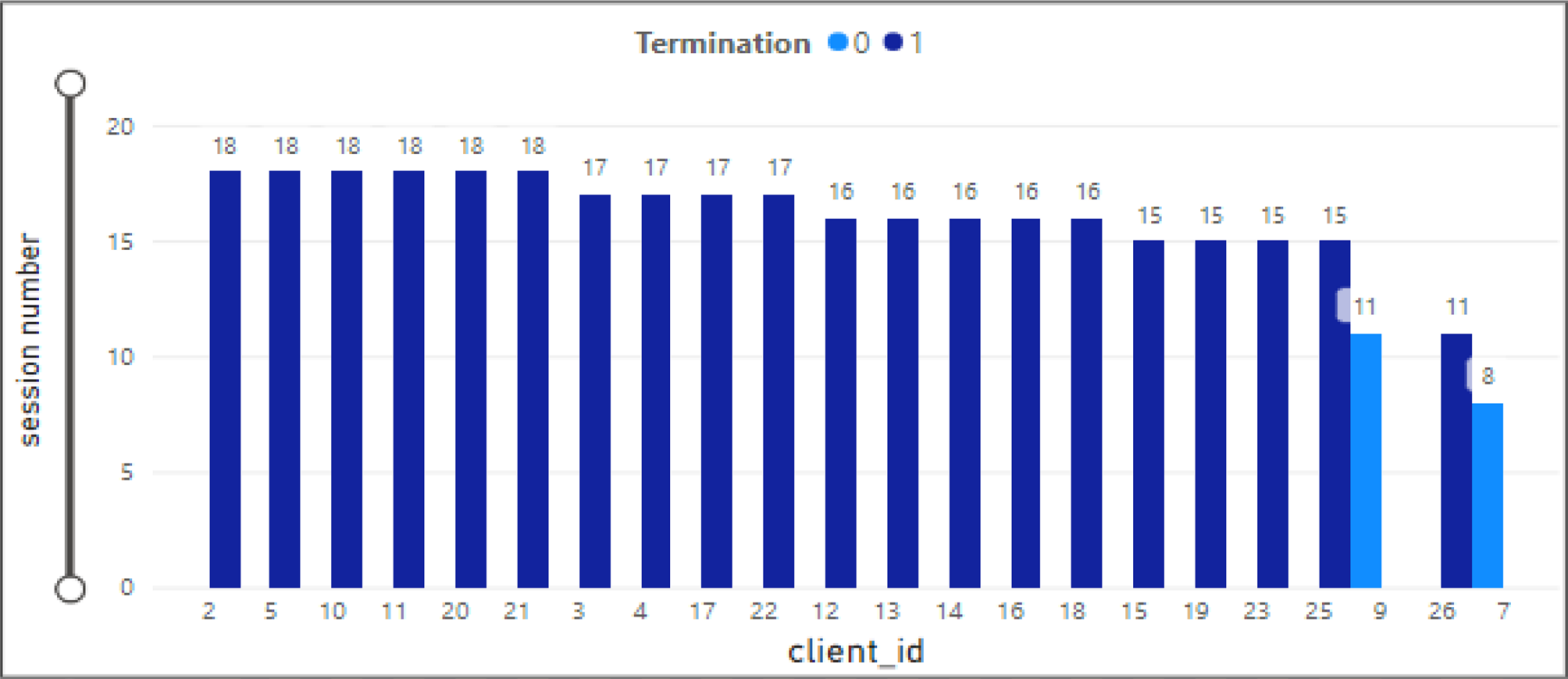
Therapy sessions attended by clients.

The bar chart in Fig. 10 presents an analysis of the total therapy sessions attended by clients. The x-axis shows twenty-two client IDs and the y-axis the total therapy sessions for each client. In addition, the legend (termination) shows if the client dropped out. The maximum number of therapy sessions (18) was observed for clients ID 11, 21, 2, 5, 10, and 20. Moreover, clients with ID number 26 only attended eleven therapy sessions. The clients with ID 9 and 7 were dropout clients (termination = 0 and attended 11 and 8 sessions, respectively).

### Sessions for therapist

**Figure 11:**
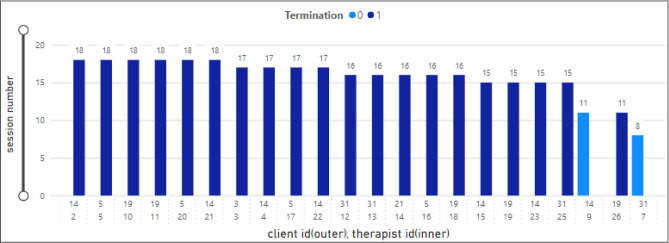
Therapy sessions attended by therapists.

Figure 11 demonstrates the same analysis for therapists. The x-axis shows therapist IDs associated with the client’s ID. The highest number of therapy sessions (18) was associated with therapists-client IDs 14-2/21, 5-5/20, 19-10/11.

### Investigating the relation of heart rate on therapeutic alliance-client

**Figure 12:**
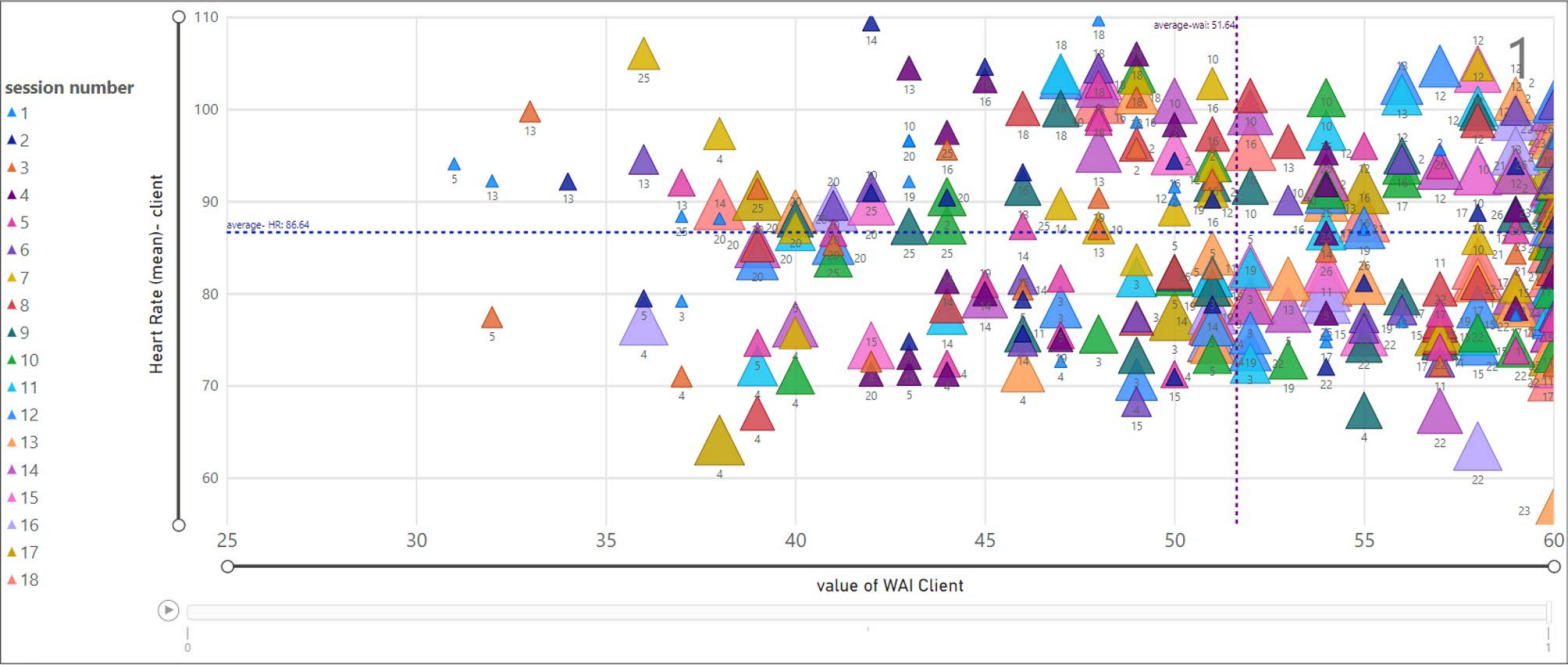
Relationship between HR and WAI score (TA) for clients; termination = 1.

Figure 12 shows the relationship between the value of TA (x-axis) and the average value of Heart Rate (HR) (y-axis) for each client (termination is equal to one). The client ID is mentioned in legend with colorful presentation and session numbers presented as data labels. Moreover, the size of each data point (triangle) depends on the session number, thus, initial sessions are presented in smaller triangles.

**Figure 13:**
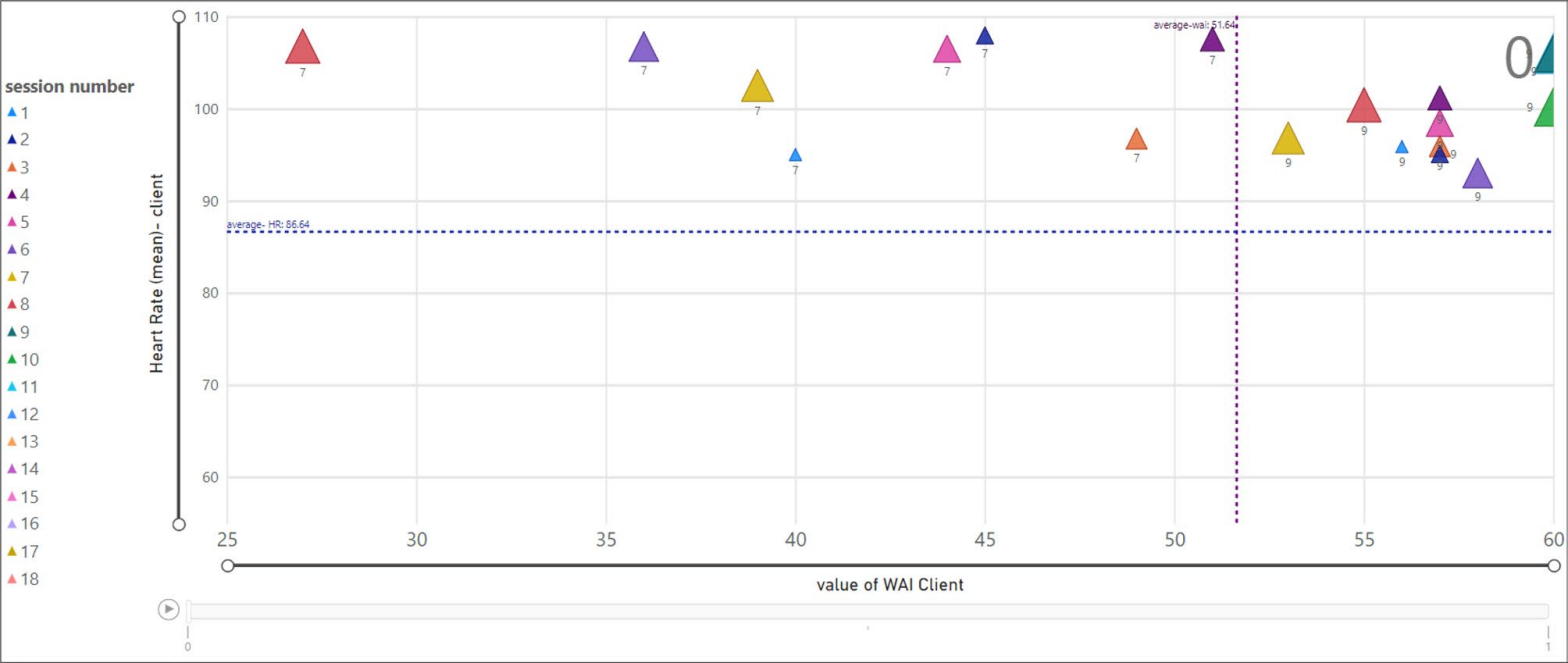
Relationship between HR and WAI score (TA) for clients; termination = 0.

Likewise, Fig. 13 presents the relationship between HR (mean) and TA (termination is zero). According to the chart, clients showed HR above the average value (86.64). The value of TA for client ID = 9 is more than the average (51.64), and for client ID = 7 is less than 51.64.

### Investigating the relation of heart rate on therapeutic alliance- therapist

**Figure 14:**
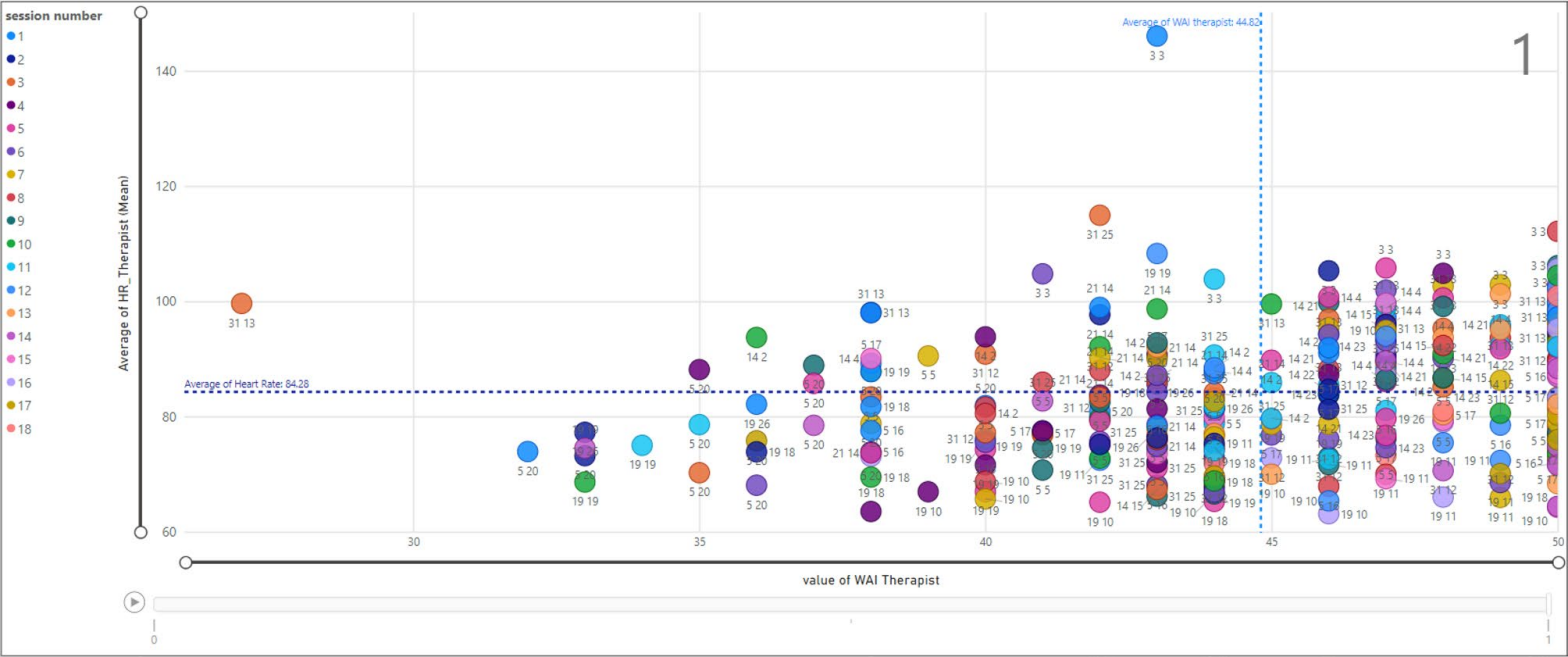
Relationship between HR and WAI score (TA) for therapists; termination = 1.

Figure 14 shows the relationship between the value of TA (x-axis) and the average value of Heart Rate (HR) (y-axis) for each therapist ID, considering that each therapist is assigned to various clients. Based on that, the marked data points show the average HR (99.64) and TA (27) in session 3 for a therapist with ID = 31 associated with a client with ID = 13. Furthermore, the vertical line presents the average value of TA for therapists which is 44.82 and the horizontal line is the average value of HR (84.28).

**Figure 15:**
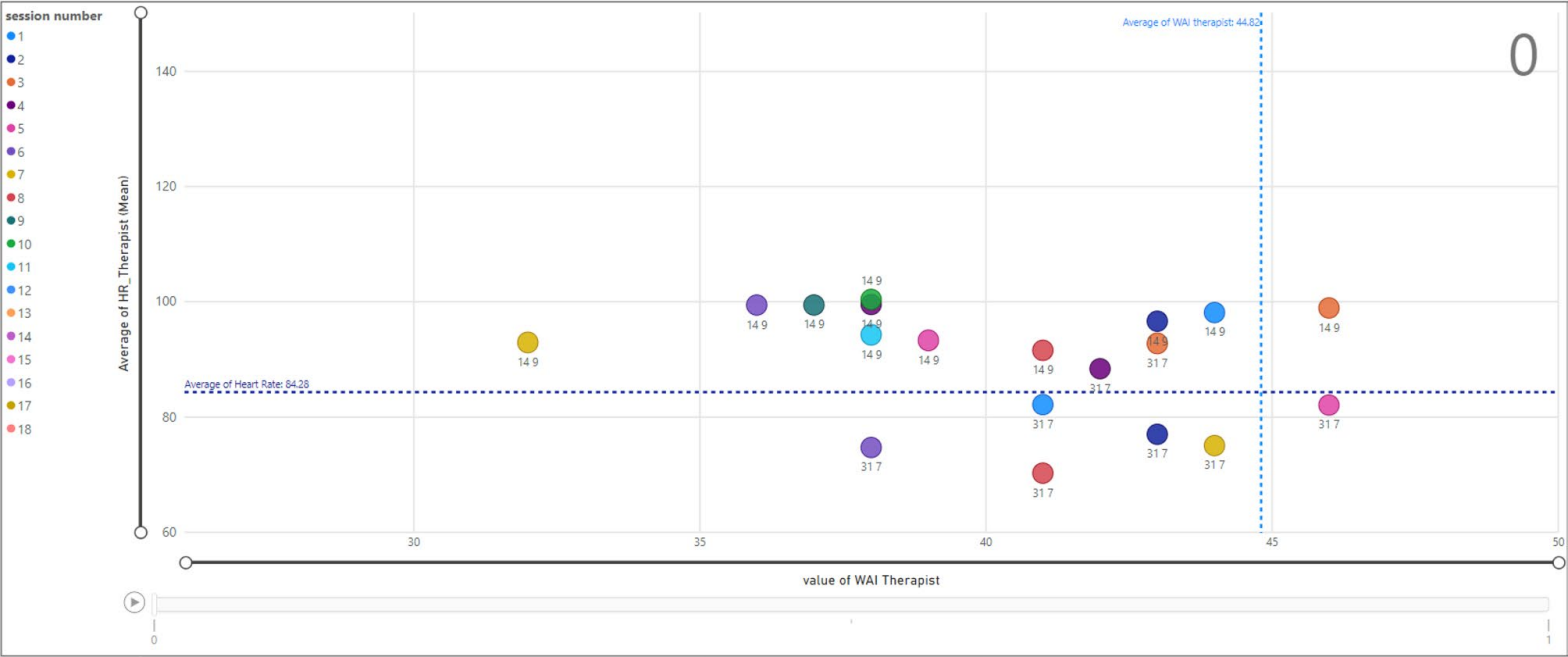
Relationship between HR and WAI score (TA) for therapists; termination = 0.

The same analysis is shown in Fig. 15 when the termination is zero. Therapist ID = 14 assigned to client ID = 9 and therapist ID = 31 associated with client ID = 7. The majority of data points are within a TA value less than average (44.82). In addition, the data points associated with therapist ID = 14 and client ID = 9 in session 1, show the average value of HR equal to 98.04 and a TA value equal to 44.

### Investigating the relation of EDA on the therapeutic alliance—client

**Figure 16:**
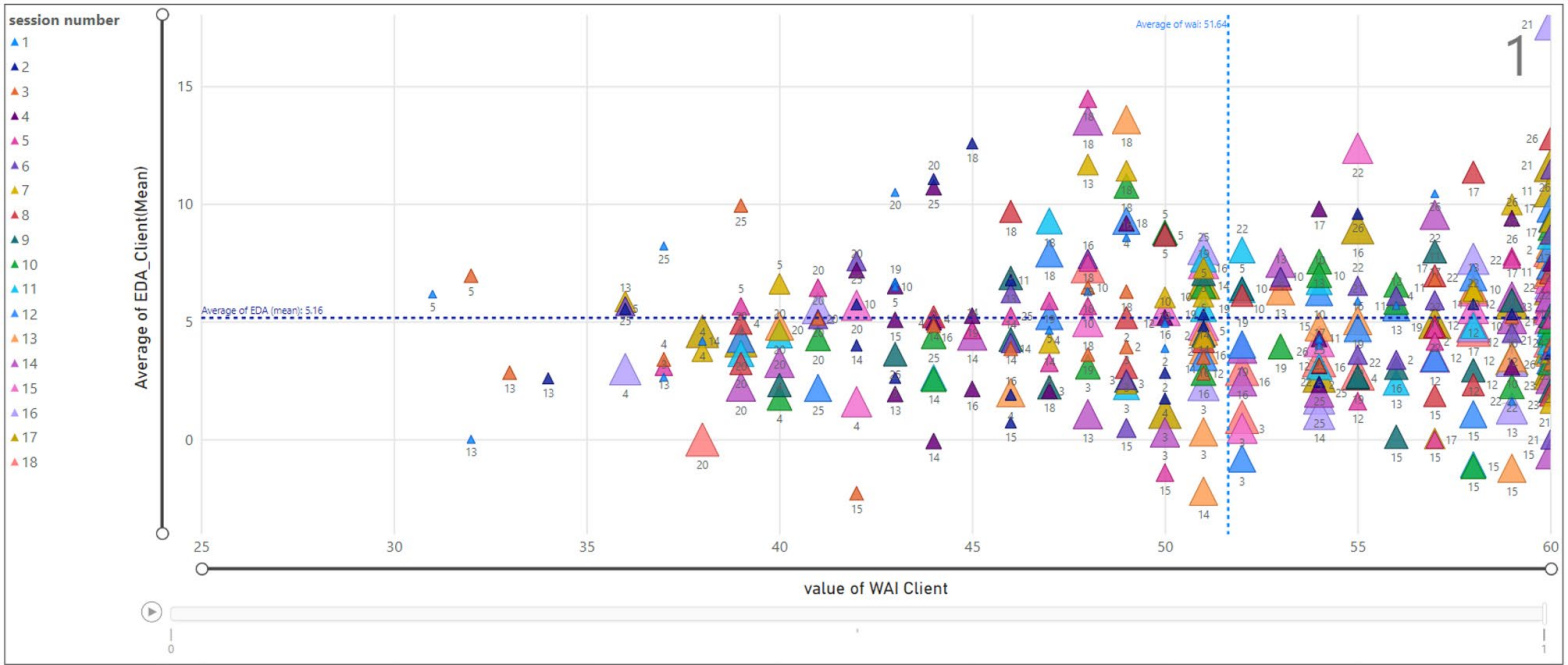
Relationship between EDA and WAI score (TA) for clients; termination = 1.

According to Fig. 16, we assessed the relationship between the TA and the average value of EDA for clients when the termination is one. The x-axis refers to the TA value and the y-axis shows the average EDA for each client and within each therapy session. The legend presents the session number with colorful triangles with the smallest triangle being associated with session one and the largest triangle with session eighteen. Moreover, the vertical average line for TA is 51.64 and the horizontal average of EDA is 5.16. An example of the selected data point displays the average of EDA (14.47) and TA value (48) for the client ID = 18 in session 5.

**Figure 17:**
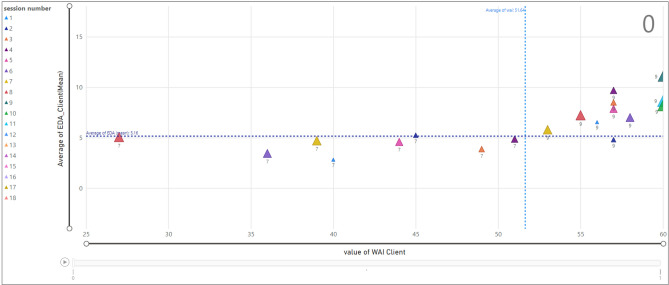
Relationship between EDA and WAI score (TA) for clients; termination = 0.

Similarly, Fig. 17 displays the same relationship when the termination is zero. According to the selected data point, client ID = 7 in session two has an average value of EDA equal to 5.25 and the TA value is 45. For example, the scatter chart shows that while the majority of data points associated with client ID = 7 carry a TA value less than average (51.64), client ID = 9 has a TA value of more than 51.64.

### Investigating the relation of EDA on the therapeutic alliance-therapist

**Figure 18:**
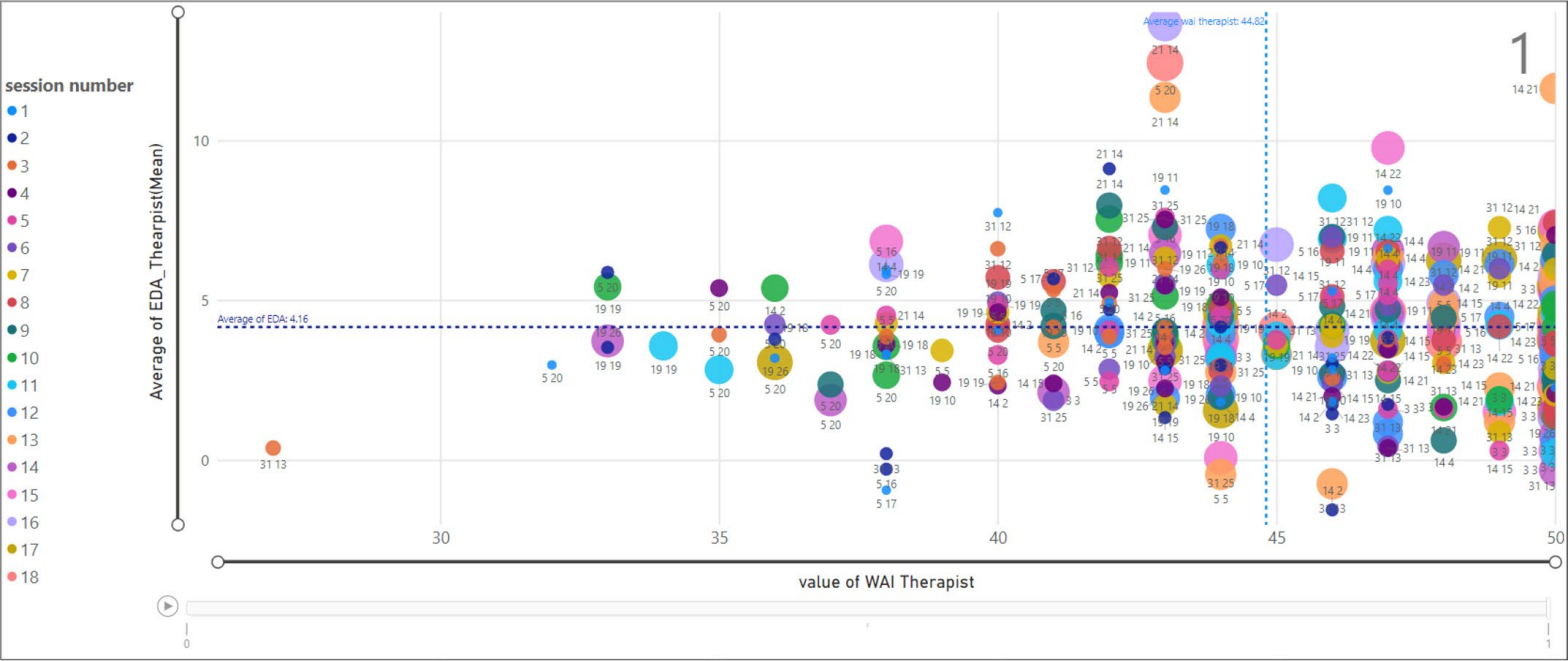
Relationship between EDA and WAI score (TA) for therapists; termination = 1.

Assessing the relationship between EDA and TA for therapists is presented in Fig. 18. The marked data point shows that the therapist ID = 21 which is assigned to client ID = 14 in session 16 has an average value of EDA equal to 13.65 and the TA value is observed as 43.

**Figure 19:**
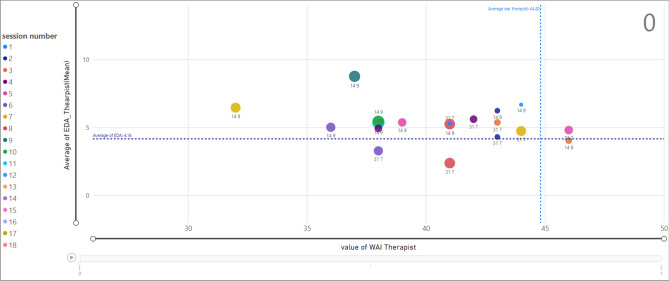
Relationship between EDA and WAI score (TA) for therapists; termination = 0.

The scatter chart in Fig. 19 shows the same analysis for termination equal to 0. The selected example presents a specific data point associated with session 10 for therapist ID =14 and client ID = 9 where the therapist EDA (mean) is 5.44 and TA value is 38.

## Data preparation

**Figure 20:**
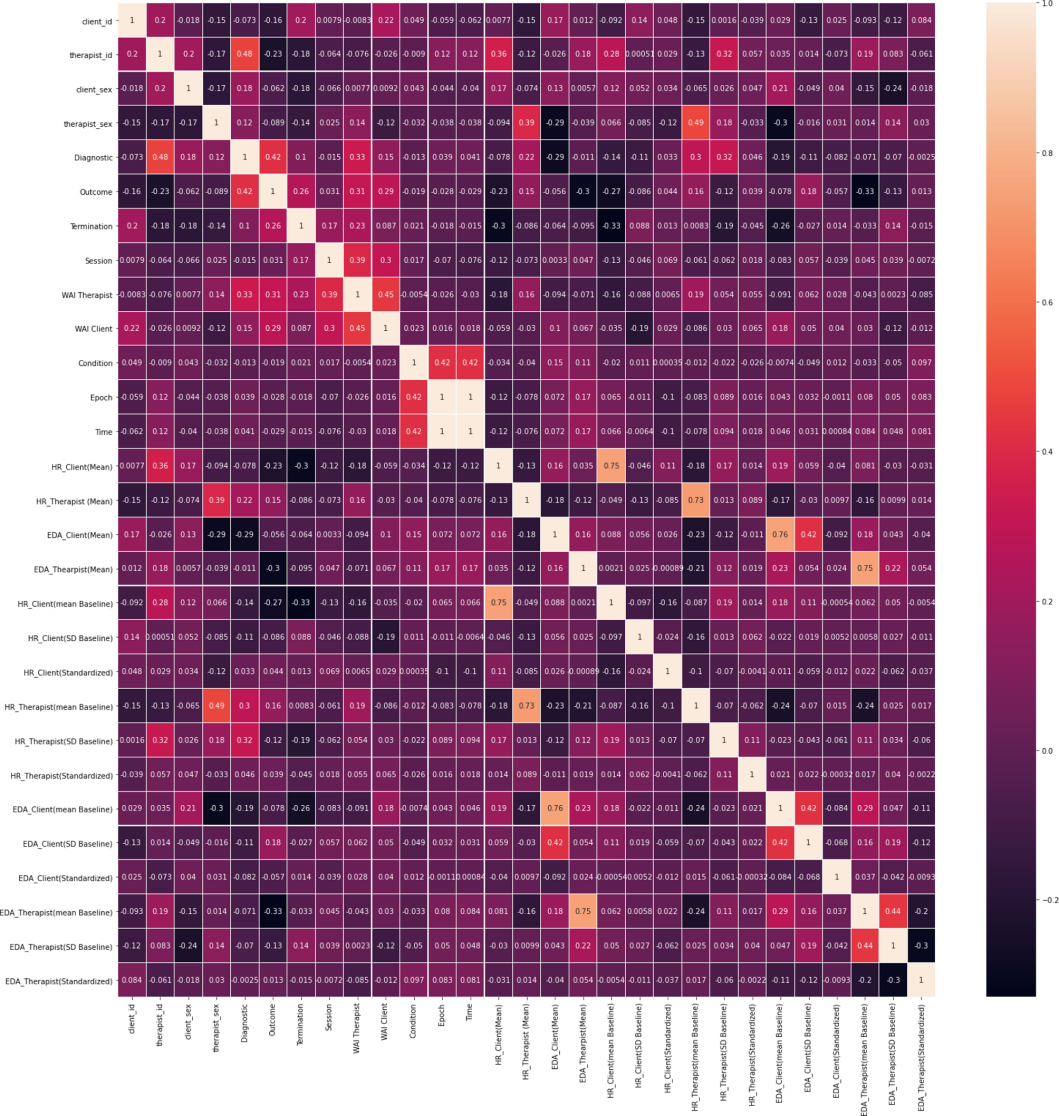
Pearson correlation coefficients matrix.

This phase consists of several tasks (e.g., feature selection, feature construction, data cleaning, and formatting)^42,43^. To prepare data for modeling, we dropped records with missing values. Furthermore, to identify the correlation coefficients between the features we have performed a Pearson correlation (i.e., “Pearson’s *r”*) that calculates the degree of linear relationship between two variables^44^. Performing correlation analysis is important to select the most influential predictors and to exclude features with a negative impact on modeling results. Additionally, since the high correlation between features is redundant and does not improve the accuracy of the models, we have excluded those features that had less influence on predicting the target (i.e., WAI-SR score). Moreover, “condition” had a constant value of 1 and was also excluded. Additionally, since the variable “ID” did not add value to the prediction model and may cause overfitting, we have excluded them. We also observed that Client_HR (Standardized) was highly skewed (Y1 = 20.54) and therefore this feature was rejected for modeling. Finally, we have changed the type of variables to the most suitable one, as some variables needed to be considered categorical, and others, as float type. Figure 20 displays the correlation matrix. According to this matrix, to identify the effective indicator for predicting the value of WAI, we considered the Pearson correlation coefficients and applied a correlation level of 0.1 <= x <= −.01. Moreover, we considered a correlation level of 0.4 <= x <= −.04 to study the relationship among variables.

Table 3 shows that the “WAI_Therapist” was the most influential indicator with a positive impact in predicting the target (correlation coefficient score of 0.44). Furthermore, “Session”, “Outcome”, “HR-Client(SD_Baseline)”, “EDA_Client(mean_Baseline)”, “Diagnostic”, “EDA_Therapist(SD_Baseline)”, “Therapist_sex” and “EDA_Client(mean)” were other effective variables in predicting the “WAI-Client” and are listed as the correlated variables to “WAI_Client”.

**Table 3:** Pearson correlation between TA (WAI Scores) and predictors.

#	Predictors for WAI_Client	score	Predictors for WAI_Therapist	Score
1	WAI therapist	0.45	WAI-Client	0.45
2	Session	0.30	Session	0.39
3	Outcome	0.28	Diagnostic	0.33
4	HR-Client(SD_Baseline)	(−) 0.18	Outcome	031
5	EDA_Client(mean_Baseline)	0.17	Termination	0.23
6	Diagnostic	0.15	HR_Therapist(mean_Baseline)	0.19
7	EDA_Therapist(SD_Baseline)	(−) 0.11	HR_Client(mean)	(−) 0.18
8	Therapist_sex	(−) 0.12	HR_Client(mean_Baseline)	(−) 0.16
9	EDA_Client(mean)	0.10	HR_Therapist(mean)	0.16

In addition, “WAI_Client” was identified as the strongest variable to predict the value of “WAI_Therapist” (correlation of 0.44). Other indicators, such as “Session”, “Diagnostic”, “Outcome”, “Termination”, “HR_Therapist(mean_Baseline)”, “HR_Client(mean)”, “HR_Client(mean_Baseline)”, “HR_Therapist(mean)” were listed as influential factors.

While EDA was not associated with “WAI_Therapist”, Heart Rate was observed as an important variable. According to Table 3, the therapist’s HR influenced WAI positively, and the Client’s HR has an inverse relation to “WAI_Therapist”. In addition, “Diagnostic” and “Termination” was more associated with “WAI_Therapist” than “WAI_Client”.

To select final predictors, we have studied the correlation coefficient among predictors. Table 4 displays the list of included and excluded variables for predicting “WAI_Client”. Considering that “session” presented a strong correlation with “WAI_Therapist '' (0.39), and “Diagnostic” with “Outcome” (0.42), these variables were excluded from modeling. Moreover, as “EDA_Client(mean)” was associated with “EDA_Client(mean_Baseline)” and had a strong link (0.75), this variable was also excluded. As referred to in the data understanding phase, because in each epoch for the same session number, the value of all EDA and HR mean_Baseline and SD_Baseline were repeated, we have aggregated data based on session number to predict WAI_Client.

**Table 4:** Pearson correlation among “WAI_Client” predictors.

#	Predictors for WAI_Client	Correlation Coefficient Score	Included	Description
1	WA therapist	0.44	Yes	
2	Session	0.30	No	High correlation with “WAI-Therapist” (0.39)
3	Outcome	0.28	Yes	
4	HR-Client(SD_Baseline)	(-) 0.18	Yes	Repeated value in each epoch for each session number
5	EDA_Client(mean_Baseline)	0.17	Yes	Repeated value in each epoch for each session number
6	Diagnostic	0.15	No	High correlation with “Outcome” (0.42)
7	EDA_Therapist(SD_Baseline)	(−) 0.11	Yes	Repeated value in each epoch for each session number
8	Therapist_sex	(−) 0.12	Yes	
9	EDA_Client(mean)	0.10	No	High correlation with “EDA_Client(mean_Baseline)” (0.75)

Analyzing the predictors of “WAI_Therapist” in Table 5, “Outcome” displayed a strong correlation with “Diagnostic” (0.42) and was excluded. In addition, to choose between “HR_Therapist(mean_Baseline)” and “HR_Therapist(mean)”, we dropped the “HR_Therapist(mean)” (with less influence on “WAI_Therapist” than the mean_baseline value.

**Table 5:** Pearson correlation Among “WAI_Therapist” predictors.

#	Predictors for WAI_Therapist	Correlation coefficient score	Included	Description
1	WAI-Client	0.44	Yes	
2	Session	0.39	No	Repeated in each epoch
3	Diagnostic	0.33	Yes	
4	Outcome	031	No	High correlation with “Diagnostic” (0.42)
5	Termination	0.23	Yes	
6	HR_Therapist(mean_Baseline)	0.19	Yes	Repeated value in each epoch for each session number
7	HR_Client(mean)	(−) 0.18	No	High correlation with “HR_Client(mean_Baseline)” (0.75)
8	HR_Client(mean_Baseline)	(−) 0.16	Yes	Choose to aggregate repeated value in each epoch for each session number
9	HR_Therapist(mean)	0.16	No	High correlation with “HR_Therapist(mean_Baseline)” (0.73)
10	Therapist_Sex	0.13	No	High correlation with “HR_Therapist(mean_Baseline)” (0.49)

HR_Client(mean_Baseline) had a correlation score of 0.75 with “HR_Client(mean)”. Thus, between “HR_Client(mean)” with negative influence (-0.18) and the “HR_client(mean_Baseline)” with a correlation equal to (-) 0.16, we chose the baseline value. We note that “HR_Therapist(mean_Baseline)” has a repeated value in each epoch for the same session. Therefore, we have aggregated data at the session level. Finally, “Therapist_Sex” was associated with “HR_Therapist(mean_Baseline)”, with a Pearson score of 0.49, and was excluded from modeling.

## Modeling

Modeling is a core step in the data mining process and includes tasks such as selecting modeling techniques, generating test designs, and assessing models^42^. In this phase, we have used the result of the correlation analysis (see 3. Data preparation) to select the most effective variables to predict the target. Based on that, to “predict WAI_Client” we have selected the top six highly correlated predictors (“WAI_Therapist”, Outcome”, Therapist_sex”, “HR-Client(SD_Baseline)”, “EDA_Client(mean_Baseline)”, “EDA_Therapist(SD_Baseline)”). Likewise, to predict the “WAI_Therapist”, we have selected as the most influential predictors the “WAI_Client”, “Diagnostic”,” Termination”, “HR_Therapist(mean_Baseline)” and “HR Client(mean_baseline)”.

In this phase, depending on the type of problem and target, various techniques use historical data for training and estimating the next event^45^. If the target is categorical, the prediction methods are called classifications; if it is continuous, it is called regression; and in the case of time-dependent targets, the prediction model is time-serious^46^.

In order to address the research question of how can data mining/ML techniques be applied to effectively predict therapeutic alliance using diverse sets of data of clients and therapists, encompassing both physiological and psychological factors we employed the most popular and promising ML methods: Artificial Neural Network (ANN), Decision Tree (DT), Random Forest (RF), Linear Regression (LR), and Support Vector Regression (SVR) algorithms. Regression is a type of Machine Learning (ML) technique that allows for delivering continuous estimates^47^. The general purpose of regression algorithms is to investigate and find the relationship between several independent variables (features, predictors) and a dependent variable or target^47^. Therefore, depending on the effectiveness and architecture of algorithms we analyzed the feature importance for the best performance algorithm to understand the influence of features in predicting the target value.

In terms of evaluating techniques, the Nested Cross Validation (CV = 5) and GridSearchCV were performed for ranking models and hyper-parameter tuning^48^. The GridSearchCV is a method in scikit-learn that automates the process of hyper-parameter tuning via exhaustively searching through a particular grid of parameter combinations. Nested cross-validation is used to evaluate and select the best model and its hyper-parameters (by GridSearchCV) in machine learning. It involves the use of two levels of cross-validation: an outer loop and an inner loop. The outer loop divides the dataset into multiple folds and iterates over them. In each iteration, one-fold is held out as a validation set, and the remaining folds are used for training. This provides an unbiased estimate of the model's performance on unseen data. The advantage of nested cross-validation is to provide a reliable estimate of the model’s performance by accounting for both hyper-parameter tuning and model evaluation^49^ and helps to avoid overfitting. The drawback of this method is that nested cross-validation is expensive in terms of computational tasks, as it requires multiple cross-validation iterations. Considering this limitation^50^ we have chosen CV = 5 folds for the inner and outer folds. Furthermore, the performance of each algorithm was evaluated by using the Mean Squared Error (MSE) (the difference between estimated and actual value), and the Coefficient of Determination (R2) (quantification of the proportion of variance in the dependent variable explained by independent variables). The less MSE, the model performs better and since R2 is the goodness of fit of a regression model, a higher value indicates better performance of the model (0 <  = R2 <  = 1)^51,52^.

While nested cross-validation focuses on assessing the overall performance and optimizing the model through hyper-parameter tuning, regression metrics such as MSE and R2 are used to evaluate the model’s predictive accuracy and how well this link is captured between features and the target value^48^.

### Decision tree (DT)

The structure of the Decision Tree Regression algorithm is based on building trees. DT divides the dataset into smaller subsets where the outcome includes decision nodes and leaf nodes. This tree-based algorithm for predicting the numeric independent variable is used to fit a sine curve with additional noisy observation^53^. In this performance, we have selected parameters such as 'max_depth' and ‘min_samples_split'.

'max_depth': [None, 5, 10] shows the maximum depth of the tree and the default value is “none”, and “min_samples_split': [2, 5, 10]” defines the minimum number of samples required to split an internal node with the default value equal to “2”^53^.

According to Table 6, the DT performed with an average MSE value of “8.04” in predicting “WAI_Client” and “16.48” in predicting “WAI_Therapist”. Furthermore, the R2 for predicting the Client’s WAI was 0.33 and the R2 for predicting the therapist's WAI was -0.23, showing a poor fitting of the DT algorithm, and operating with less error over the client’s data.

**Table 6:** Performance of DT.

Evaluation of DT			
Metrics(average)	Client	Therapist	Parameters
MSE	8.04	16.48	'max_depth': [None, 5, 10], 'min_samples_split': ^2,5,10^
R2	0.33	(−) 0.23	

### Support vector regression (SVR)

SVR is a powerful algorithm that gives the flexibility to choose the tolerance of errors, both through an acceptable error margin (ϵ) and through tuning our tolerance of falling outside that acceptable error rate. SVR performs by finding a line of best fit that minimizes the error of a cost function and uses a C parameter, called the complexity parameter, which controls how flexible the process for drawing the line to fit the data (“C':[1, 10, 100]), “kernel': ('linear', 'rbf')” (type of kernel)^54^.

Table 7 shows that the average value of MSE was 5.79 for predicting the client's WAI, and 9.15 for predicting the therapist’s WAI. Moreover, the good score of the client’s R2 (0.54) compared to the therapist’s R2 (0.17) shows that the SVR displayed better results for predicting clients’ WAI than the therapist’s WAI.

**Table 7:** Performance of SVR.

Evaluation of SVR			
Metrics (average)	Client	Therapist	Parameters
MSE	5.79	9.15	'C': [1, 10, 100], 'kernel': ['linear', 'rbf']
R2	0.54	0.17	

### Random forest (RF)

RF fits several decision trees on various sub-samples of the dataset and uses averaging to improve the predictive accuracy and control overfitting^55^. This technique increases the diversity of the trees by creating them from various training data subsets. This procedure is called bagging^56^. GridSerachCV chooses the best combination of given parameters (max_depth, and n_estimators) for obtaining the optimized result.

“max_depth': [100, 200, 300]” refers to the maximum depth of the tree, and n_estimators': [10, 100, 500]' ' defines the number of decision trees in the forest. Increasing the number of estimators can improve the model's performance, but it also increases computation time^57^.

According to Table 8, the average value of MSE for the client’s WAI was 5.49, and for the therapist’s WAI was - 9.56. Considering the client’s R2 (0.59) in comparison with the therapist’s R2 (0.16), we observed that RF performed with a lower error rate to predict the client’s WAI.

**Table 8:** Performance of RF.

Evaluation of RF			
Metrics (average)	Client	Therapist	Parameters
MSE	5.49	9.56	n_estimators': [100, 200, 300], 'max_depth': [None, 5, 10]
R2	0.59	0.16	

### K nearest neighbor regression (KNN)

In this algorithm, the input consists of the *k-closest* training set. The output value is the average of the values of *KNN*. If *k* = 1, then the output is assigned to the value of that single nearest neighbor^58^.

Table 9 shows the result of the KNN performance and the list of parameters (n_neighbors, weights). The “n_neighbors^3,5,7^” refers to the number of neighbors to be used, which by default is 5. The weights: ['uniform', 'distance']” is used for prediction. “Uniform” is the default and means that all points in each neighborhood are weighted equally. In “distance”, weight points by the inverse of their distance^59^.

**Table 9:** Performance of KNN.

Evaluation of KNN			
Metrics (average)	Client	Therapist	Parameters
MSE	7.59	9.88	'n_neighbors': [3, 5, 7], 'weights': ['uniform', 'distance']
R2	0.42	0.20	

Assessing the KNN model, the average value of MSE was 7.59 for predicting the client’s WAI and 9.88 for the therapist’s WAI. Furthermore, observing the client’s R2 (0.42) and therapist’s (0.20), we can observe that KNN performed poorly in predicting the therapist’s WAI in comparison with the client’s WAI.

### Linear regression (LR)

LR is a predictive method to identify the link among the variables when there is a linear relationship between them^60^. This method fits a linear model with coefficients to minimize the residual sum of squares between the observed targets in the dataset and the predicted target by LR^61^.

In linear regression, there are no specific hyper-parameters to tune like in other techniques. Data pre-processing aspects (e.g., handling missing values and outliers) are important steps to optimize the achieved result. In addition, using metrics such as MSE and cross-validation methods is helpful to obtain a reliable outcome^62^.

The “fit_intercept': ['True',' False'] parameter is used to calculate the intercept for this model. If false, no intercept will be used in calculations (i.e., data is expected to be centered). Moreover, in the context of scikit-learn’s LR model, “'copy_X': ['True', 'False']” determines whether to make a copy of input features (X) before the fitting process. Setting this parameter to “False” we can save memory. Based on the Nested cross validation’s outcome, the GridSearchCV chose “True” for both parameters which is the default value too.

According to Table 10, LR was more effective in predicting the client’s WAI than the therapist’s WAI. The average value of MSE to predict the client’s was 6.06, and the R2 was o 0.53, showing a good fit. This value (average of MSE) for the therapist’s WAI was 7.90 and the therapist’s R2 was 0.30.

**Table 10:** Performance of LR.

Evaluation of LR			
Metrics (average)	Client	Therapist	Parameters
MSE	6.06	7.90	fit_intercept': ['True',' False'], 'copy_X': ['True', 'False']
R2	0.53	0.30	

### Artificial neural network (ANN)

ANN ML is a multilayer perceptron regressor. This optimizes the squared error using LBFGS or stochastic gradient descent. The architecture of this technique consists of an input and an output layer to present and obtain data. The parameters of the ANN, such as the choice of input nodes, number of hidden layers, number of hidden nodes (in each hidden layer), and the form of transfer functions, depending on the type of problem and achieving the best performance of the model requires trial and error^63^.

Table 11 shows the performance of ANN and the parameters used in this algorithm. The average value of MSE for the client’s WAI is equal to 6.42 and R2 is 0.46. Furthermore, the average value of MSE to predict the therapist’s WAI is equal to 8.51, and the R2 is 0.14.

**Table 11:** Performance of ANN.

Evaluation of ANN			
Metrics (average)	Client	Therapist	Parameters
MSE	6.42	8.51	'hidden_layer_sizes': [(10,), (20,), (30,)], 'activation': ['relu', 'tanh'], 'solver': ['adam', 'lbfgs']
R2	0.46	0.14	

Although there are various parameters to run the ANN, we applied GridSearchCV to choose the best combination of 'hidden_layer_sizes': [(10,), (20,), (30,)] representing the number of neurons in the hidden layer with default = 100) 'activation': ['relu', 'tanh'] which refers to the activation function for the hidden layer (‘‘tanh’, the hyperbolic tan function, returns f(x) = tanh(x), ‘relu’, the rectified linear unit function, returns f(x) = max (0, x). 'solver': ['adam', 'lbfgs'] to optimize the weight (‘lbfgs’ is an optimizer in the family of quasi-Newton methods, ‘adam’ refers to a stochastic gradient-based optimizer)^63,64^.

Overall, based on the performance of the above-mentioned algorithms, we observed that the LR and RF were identified as the most adjusted techniques to predict the WAI (for clients and therapists). Therefore, to investigate the level of the feature’s influences in predicting the target value, LR uses coefficient implementation, which employs the weighted sum to make a prediction. These coefficients can be used directly as a crude type of feature importance score. In addition, in RF, the feature importance indicates the relative importance of each predictor.

In Table 12, we show the results of both techniques (LR and RF) in predicting the therapist’s and client’s WAI. For the therapist's WAI, the “Diagnostic” (score = 3.62) and “Termination” (score = 2.93) were ranked as the most effective variables with positive influence. Whereas the “HR_Client(Mean_Baseline)” was less influential, the “HR_therapist(mean_baseline) and HR_client(mean_baseline)” influenced the therapist’s WAI. Specifically, less HR(mean_baseline) in both clients and therapists resulted in more therapist WAI. HR_therapist(mean_baseline) was also identified as an important physiological indicator, with a coefficient score of (−) 0.15.

**Table 12:** Feature importance by LR and RF.

Feature importance to predict Therapist’sWAI by LR	Scores	Feature importance to predict Client’sWAI by RF	Scores
Diagnostic	3.62	WAI Therapist	0.4072
Termination	2.93	Outcome	0.3161
WAI Client	0.20	EDA_Client(mean Baseline)	0.1377
HR_Therapist(mean_baseline)	− 0.15	HR_Client(SD Baseline)	0.0614
HR_Client(mean_baseline)	− 0.02	EDA_Therapist(SD Baseline)	0.0609
NA	NA	therapist_sex	0.0165

For the client's WAI, we observed that the therapist’s WAI (score = 0.40) and “outcome” (score = 0.31) were significant predictors. In terms of physiological indicators, the EDA_client (mean baseline) (score = 0.13) was identified as a significant physiological indicator.

In conclusion, in terms of physiological indicators, while Heart Rate(mean_baseline) was an important physiological factor to predict the therapist’s WAI (with negative impact), the EDA(mean_baseline) was observed as an influential indicator to predict the client’s WAI.

## Evaluation

Whereas in the modeling phase, both the accuracy and generality of the model are assessed, in the evaluation phase we illustrated to which degree the model meets the objectives. In other words, we evaluated the suitability of the model by considering the application objectives. This level includes three tasks: evaluation, review, and deciding which algorithm meets the objectives. The final decision defines whether the selected model will be deployed or not^53^.

Considering the results obtained in the modeling phase, we can observe the comparison of techniques concerning the data mining goals (see Table 13). The evaluation of the six regression algorithms showed that the Linear Regression technique was the most competitive technique to predict the therapist’s WAI. The average of MSE was 7.90 and the average of R2 was 0.30. Observing the LR as the most ranked algorithm to predict the therapist’s WAI shows that there is a linear relation identified by the model, although the value of R2 highlights that this linear link between predictors and the target is not significant. Based on that, the coefficient score displays the variables “Diagnostics” (coefficient = 3.62) and “Termination” (coefficient = 2.93) as the most effective predictors. Moreover, the “HR _therapist(mean_baseline) was identified as the physiological predictor, having a negative impact on the therapist’s WAI (coefficient = -0.15).

**Table 13:** Ranking techniques with significant predictors.

Technique	Client’sWAI		Therapist’s WAI		Top predictor for Client’sWAI by RF	Top Predictors for therapist’s WAI by LR
	MSE	R2	MSE	R2	Physiological|NOT	physiological|NOT
ANN	6.42	0.46	8.51	0.14	physiological: EDA_Client(mean Baseline) score:0.13	physiological: HR_Therapist (mean_baseline) score: (−) 0.15
RF	5.49	0.59	9.66	0.16		
DT	8.04	0.33	16.48	(-) 0.23		
KNN	7.59	0.42	9.88	0.20	Not physiological:therapist’s WAI; score; 0.41 Outcome; score: 0.31	Not physiological: Diagnostic; score: 3.62 Termination; score:2.93
SVR	5.79	0.54	9.15	0.17		
LR	6.06	0.53	7.90	0.30		

To predict the Client’s WAI, the Random Forest has shown to have the best possible performance, with MSE of 5.49 and a R2 of 0.59. According to the RF’s feature importance implementation, higher scores represent greater importance. Thus, “Outcome” and “therapist_WAI'' were the two top indicators to predict a client’s WAI. Regarding the impact of physiological factors, clients’ EDA (mean_baseline) affected the estimation of the client’s WAI.

## Discussion

In this study, we have applied data mining techniques via CRISP-DM methodology to understand the quality of the therapeutic alliance in both clients and therapists, considered as a micro-outcome at the therapy session level, in which variables like session, outcome, diagnostic, termination, sex, HR, and EDA. Although in the data understanding phase (data analysis) we did not observe any particular relationship between different variables and TA, the use of ML techniques provided new insights and identified the variables that influenced the quality of TA. According to the outcome of nested cross-validation that ranked the performance of regression algorithms (RF, DT, LR, SVM, ANN, and KNN), we observed that RF was the algorithm that achieved the best performance for predicting the client’s TA. In particular, therapy “Outcome” and “Therapist’s WAI” were the most relevant indicators to predict the client’s TA. Furthermore, to predict the therapist’s TA, the LR has emerged as the best algorithm, identifying a linear link between client “Diagnostic”, therapy “Termination” and the therapist’s TA.

Additionally, the result of modeling showed that, while there was a linear relationship between the therapist’s HR (Mean_Baseline) and the perception of the therapist’s TA, i.e., lower HR at baseline resulted in better scoring of the WAI-SR, this linear relationship was not applicable for clients. Moreover, we did not observe any linear relationship between EDA and TA for both clients and therapists^64^. With this study approach, we provided evidence that ML tools proved to be useful for knowledge discovery in the field of psychotherapy, as others have previously demonstrated^29,30^.

Overall, results from the modeling phase of the CRISP-DM suggested that lower HR is related to the therapist’s perception of a good interpersonal therapy experience (i.e., collaborative work, agreement on goals and therapy tasks, and a good bond quality with the therapist). Although HR is controlled by both branches of the autonomic nervous system (parasympathetic nervous system and sympathetic nervous system)^65^, evidence suggests that lower HR is likely to be more influenced by the parasympathetic branches and cardiac vagal activity due to what is called “accentuated antagonism” mechanisms^66,67^. Cardiac vagal regulation (e.g., measured through HRV measures) has been proposed as a pivotal neurophysiological mechanism for social engagement^68^ by either (a): reducing cardiac output enabling us to rapidly self-soothe and regulating our visceral state and fostering engagement with other individuals^69^; (b) increasing cardiac output and producing mobilization behaviors, thus avoiding social involvement^70^.

Therefore, we hypothesize that the therapist’s lower HR at baseline may constitute a surrogate biological marker of comfort, fostering the client’s caring and the willingness to align and affiliate with the client's needs, communicate and foster feelings of safety in a therapeutic context thus enabling a deeper engagement with therapy work that will further have an impact on the therapist’s perception of the alliance at the end of each session. It is possible to lower HR at baseline might promote co-regulation and facilitate cooperation between the therapeutic dyad. Although using different physiological cardiac measures (i.e., HR in our study), our results seem to be consistent with the empirical research suggesting that lower levels of stress during therapy promote the development of successful therapeutic alliances^24,71–73^. In this line, studies have documented HRV as an index of interpersonal interaction in the context of therapy, being associated with the therapeutic alliance^24,72^. Specifically, lower HRV has been associated with decreased cognitive and emotional regulation, and higher HRV (more vagal modulation) with increased social interaction skills^74^. Other studies have found that higher levels of clients’ perception of the therapeutic alliance at the end of therapy sessions were related to lower levels of client HRV^24^ and increased clients’ in-session high-frequency HRV^23^. Finally, higher levels of HRV have been also associated with increased perceived social support in contexts of stress-related experiences^75^. Overall, these results highlight the role of cardiac autonomic activity concerning TA and are indirectly supported by our results, showing the impact of the HR (mean_Baseline) variable in the TA, as demonstrated also by the LR algorithm.

Regarding the RF results, EDA emerged as the most influential biological feature in the prediction of the target – TA, in the client. EDA has been considered an index of emotional regulation and empathic response, as it reflects a sensitive measure of emotional arousal in social interaction and sympathetic activation^17^. Curiously, our results did not show this pattern, when the model was explored for the therapist’s TA. Considering studies document that increased EDA synchrony between client and therapist has been related to increased empathy and therapeutic alliance^17,19,26^, we would expect that the therapist and the client’s emotional co-regulation, translated through physiological signals (EDA), would influence both perspectives of TA (client and therapist). However, our model did not consider EDA physiological synchrony between the dyad, but our RF results suggest EDA may be considered in future studies under an interpersonal physiological synchrony framework. Furthermore, it is also possible that clients who are emotionally more regulated before sessions are willing to mutually engage with the therapist and the therapy work, thereby being more open to the benefits of the therapist's empathic and caring interventions, which in turn, may impact the alliance evaluation^20^.

Overall, the results from the ML algorithm document the differential importance of the physiological variables in the therapist and client (HR and EDA, respectively), for predicting TA, suggesting different experiences during therapy sessions for the dyad and with different underlying neurophysiological mechanisms^23^. One possibility is that the therapist uses a more selective and focused strategy to evaluate the quality of the collaborative work during the session, i.e., the therapist’s perception of alliance tends to be more technical, theoretically based, and estimated by reference to other clients, clients tend to use their other meaningful relationships as reference^9,23^. Thus, clients and therapists differ in the main strategies to assess TA and rely on different predictors (i.e., feature importance in ML), which is in agreement with others^23^. This is consistent with our physiological results (HR for therapist and EDA for client), suggesting that clients and therapists may use different baseline and in-session physiological states to evaluate the quality of alliance.

Our results also strongly support the pivotal relationship between therapeutic alliance and therapy outcomes, as widely documented^76^, namely when the alliance was evaluated by clients. It is interesting to find that therapist alliance evaluation is also a predictor of the client’s alliance evaluation, suggesting a convergence or synchrony between the dyad. Additionally, “Diagnostic” is an important feature to predict the target (TA), suggesting that a major depression or social anxiety diagnosis has an impact on the way TA is assessed by the therapist. There is mixed evidence on whether the client diagnosis influences alliance ratings^6,8^. While some studies have shown that the assessment of TA is a transdiagnostic phenomenon^8,77^, other evidence states that diagnosis may affect the agreement of clients' and therapists’ perspectives on TA^2^. Although our study did not address how convergent the client-therapist estimates of TA were according to diagnosis, we know that pretreatment expectations toward change and the severity of interpersonal problems influence TA evaluation at the beginning and throughout the therapy process^2,78^. Therefore, it is likely that these factors are differentially expressed in social anxiety and major depression, and therefore are contributing to the influential role of diagnosis in predicting therapist evaluation of TA observed in this study. While our results are in line with research indicating a discrepancy between therapist and clients’ perspectives on alliance evaluation^9^, other studies have shown convergence between both perspectives on the evaluations of alliance throughout the therapy process from the psychological point of view^76^.

Furthermore, the outcomes of this work, suggest that different factors are relevant in predicting therapist and clients alliance evaluation, with clients being influenced by progress in therapy and the therapist's perception of their collaboration, and therapist being influenced by the client’s diagnosis and the client's maintenance or dropping out of therapy (probably manifested on their engagement on therapy). In line with the growing number of studies on physiological synchrony in psychotherapy^79^, it would be interesting to study the convergence of therapist and client physiological measures, such as HR or other measures of physical experience through therapy, and its relation with convergence on the alliance ratings.

As a clinical implication of this research, it is pivotal to understand the multidimensionality of the client’s and therapist's interactional processes. Taking physiological reactivity as a barometer of the quality of therapeutic alliance may help the therapists to responsively adjust their interventions to the client's actions, for example paying attention to certain social cues like touch, voice tone, facial expressions, and employing behaviors that communicate and foster feelings of safety and contribute to regulate physiological activity. Therefore, this study contributes to highlighting the relevance of the therapists' competence in being aware of their own internal experiences as well as to the client's body manifestations of their internal experiences in the context of the interpersonal process within therapy sessions. Future studies on the relationship between physiological variables such as HR and EDA and observable body manifestations of both therapists and clients in the naturalistic context of therapy would help to elaborate on the clinical implications of this study.

This study brings essential insights into the use of data mining in psychotherapy. The data mining approach and ML are recent and increasingly used techniques in psychotherapy to support clinical decision-making by considering patients' characteristics, history of treatment, and other relevant factors^80^. Moreover, these algorithms are capable of capturing intricate patterns and relationships, enabling more accurate predictions of the therapeutic alliance. However, to our knowledge, such technologies were rarely used in the area of therapeutic alliance^31,34^. We have therefore shown that these techniques can be employed to strengthen TA. Prediction models can be trained and learn complex relationships and predict the level or quality of therapeutic alliance based on a set of input variables and patterns identified in the data. Additionally, by integrating significant amounts of data (biological, psychological) we may understand, monitor, and predict the outcomes of the client’s psychotherapeutic process. In fact, machine learning models excel at tailoring predictions based on the unique characteristics of individual clients and therapists. By analyzing specific features and their interactions, machine learning provides personalized insights into the factors that contribute to a strong therapeutic alliance. This personalized approach empowers clinicians to deliver customized interventions and enhance the effectiveness of the therapeutic process^27^. Furthermore, the complexity and non-linear relationships within this data can be effectively addressed using machine learning algorithms^81–83^.

Accounting for such techniques in psychological treatment and psychotherapy research has not been systematic. However, as documented by others, they can valuably complement the traditional regression models^84^ and introduce novelty to studying important psychotherapy concepts^29,31^. Specifically, in the scope of the therapeutic alliance, ML allows: (a) knowledge discovery via identifying the possible relationships between variables (i.e., physiological factors, outcome, termination, demographics, number of sessions, among others); (b) analyze data collected from multiple therapy sessions and tracking the changes in the client’s outcome, as data mining can provide insight into how alliance contributes to treatment success and failure; (c) predictive modeling, as data mining is a powerful way to enable the development of predictive models associated with the quality of therapeutic alliance, as ML algorithms use historical data to train and predict the likelihood of a positive alliance based on the various factors; (d) to support evidence-based practice by providing empirical evidence and identify effective predictors that influence the TA and serve as guidelines for fostering a positive therapeutic alliance; (e) to determine individual differences and their impact on the therapeutic alliance is an important step toward personalizing the treatment.

Even though the present study has potential, some limitations should be mentioned. Psychotherapy is a complex interpersonal process, and relational and other concurrent factors are difficult to disentangle. As above mentioned, this was an exploratory study using Data Mining/ ML to different therapist and client variables during different phases of the session. Therefore, although the model was able to establish a predictive relation between the physiological and psychological data (input) and the TA (target), they should not be interpreted as a “cause” and “effect” relationship, as it is likely that bidirectional associations between variables may exist, or interactions of predictors with different therapeutic processes. In fact, the assessment of therapeutic alliance is affected by processes such as safety, engagement, coregulation, and cooperation, all interpersonal experiences that affect and are affected by physiological mechanisms, as documented by several studies^17–20,23,24^.

We recognize that the identification of causal explanations or predictions in psychotherapy is a challenging issue, and has been subject to intense debate in different psychotherapy contexts (see for example studies in EMDR on the identification of physiological mechanisms or predictors associated with treatment outcome)^85^, including those using ML techniques. Therefore, future studies could apply other techniques and experimental designs to assess other variables associated with TA and to what extent the different physiological measures/analyses are driven by TA, to better clarify the phenomena. The clients who participated in this study were diagnosed with major depression or social anxiety, sometimes with comorbidity, which may have had an impact on HR measures. While the therapeutic alliance is a pan-theoretical concept, since all the clients were treated in cognitive behavior therapy, which is characterized by a greater structure in the interpersonal behavior and therapy tasks than other therapy approaches, generalizations should be cautious. In addition, our study has some limitations regarding the different physiological measures, as we cannot extend our results to other studies that have used HRV or a synchrony-based approach. Furthermore, even though we have used wireless electrodes, these artificial procedures interfere with a naturalist context such as psychotherapy.

## Data Availability

Data has been uploaded to the public repository; Kaggle. @misc{nasim sadat mosavi_2023, title={therapeutic alliance_ clients and therapists}, url={https://www.kaggle.com/dsv/6168984}, DOI={10.34740/KAGGLE/DSV/6168984}, publisher={Kaggle}, author={Nasim Sadat Mosavi}, year={2023}}.

## References

[1] Lutz W, Castonguay L, Michael L, Barkham M, Barkamn, Lutz, Castonguay (2021). Traditions and new beginnings: Historical and current perspectives on research in psychotherapy and behavior change. Bergin and Garfields, Handbook of Psychotherapy and Behavior Change.

[2] Crits-Christoph P, Gibbons MBC, Barkamn, Lutz, Castonguay (2021). Psychotherapy process-outcome research: Advances in understanding causal connections. Bergin and Garfields, Handbook of Psychotherapy and Behavior Change.

[3] Horvath AO, Luborsky L (1993). The role of the therapeutic alliance in psychotherapy. J. Consult. Clin. Psychol..

[4] Norcross JC, Wampold BE (2018). A new therapy for each patient: Evidence-based relationships and responsiveness. J. Clin. Psychol..

[5] Del Re AC, Flückiger C, Horvath AO, Wampold BE (2021). Examining therapist effects in the alliance–outcome relationship: A multilevel meta-analysis. J. Consult. Clin. Psychol..

[6] Flückiger C (2020). Assessing the alliance–outcome association adjusted for patient characteristics and treatment processes: A meta-analytic summary of direct comparisons. J. Couns. Psychol..

[7] Zilcha-Mano S, Fisher H (2022). Distinct roles of state-like and trait-like patient—Therapist alliance in psychotherapy. Nature Rev. Psychol..

[8] Igra L (2020). A meta-analysis of client-therapist perspectives on the therapeutic alliance: Examining the moderating role of type of measurement and diagnosis. Eur. Psychiatry.

[9] Tryon GS, Blackwell SC, Hammel EF (2007). A meta-analytic examination of client—Therapist perspectives of the working alliance. Psychother. Res..

[10] Jennissen S, Nikendei C, Ehrenthal JC, Schauenburg H, Dinger U (2020). Influence of patient and therapist agreement and disagreement about their alliance on symptom severity over the course of treatment: A response surface analysis. J. Couns. Psychol..

[11] Laws HB (2017). Convergence in patient–therapist therapeutic alliance ratings and its relation to outcome in chronic depression treatment. Psychotherapy Res..

[12] Muntigl P, Scarvaglieri C (2023). Discursive angles on the relationship in psychotherapy. Front. Psychol..

[13] Heinonen E (2014). Therapists’ professional and personal characteristics as predictors of working alliance in short-term and long-term psychotherapies. Clin. Psychol. Psychother..

[14] Tschuschke V, Koemeda-Lutz M, von Wyl A, Crameri A, Schulthess P (2022). The impact of clients’ and therapists’ characteristics on therapeutic alliance and outcome. J. Contemp. Psychother..

[15] Wampold BE, Baldwin SA, Holtforth MG, Imel ZE, Gastonguay LG, Hill CE (2017). What characterizes effective therapists?. How and Why are Some Therapists Better than Others? Understanding Therapist Effects.

[16] Uckelstam C-J, Holmqvist R, Philips B, Falkenström F (2020). A relational perspective on the association between working alliance and treatment outcome. Psychother. Res..

[17] Marci CD, Ham J, Moran E, Orr SP (2007). Physiologic correlates of perceived therapist empathy and social-emotional process during psychotherapy. J. Nerv. Ment. Dis..

[18] Riess H (2011). Biomarkers in the psychotherapeutic relationship: The role of physiology, neurobiology, and biological correlates of EMPATHY. Harv. Rev. Psychiatry.

[19] Tourunen A (2020). Sympathetic nervous system synchrony: An exploratory study of its relationship with the therapeutic alliance and outcome in couple therapy. Psychotherapy.

[20] Doukas A, D’Andrea W, Doran J, Pole N (2014). Psychophysiological predictors of working alliance among treatment-seeking women with complex trauma exposure. J. Trauma Stress.

[21] Del Piccolo L, Finset A (2018). Patients’ autonomic activation during clinical interaction: A review of empirical studies. Patient. Educ. Couns.

[22] Voutilainen L (2018). Empathy, challenge, and psychophysiological activation in therapist-client interaction. Front. Psychol..

[23] Blanck P, Stoffel M, Bents H, Ditzen B, Mander J (2019). Heart rate variability in individual psychotherapy. J. Nerv. Ment. Dis..

[24] Stratford T, Lal S, Meara A (2012). Neuroanalysis of therapeutic alliance in the symptomatically anxious: The physiological connection revealed between therapist and client. Am. J. Psychother..

[25] Heaphy ED, Dutton JE (2008). Positive social interactions and the human body at work: Linking organizations and physiology. Acad. Manag. Rev..

[26] Bar-Kalifa E (2019). Physiological synchrony and therapeutic alliance in an imagery-based treatment. J. Couns. Psychol..

[27] Becker D (2018). Predictive modeling in e-mental health: A common language framework. Internet Interv..

[28] Shatte ABR, Hutchinson DM, Teague SJ (2019). Machine learning in mental health: A scoping review of methods and applications. Psychol. Med..

[29] Aafjes-van Doorn K, Kamsteeg C, Bate J, Aafjes M (2021). A scoping review of machine learning in psychotherapy research. Psychother. Res..

[30] Rollmann I (2023). Systematic review of machine learning utilization within outpatient psychodynamic psychotherapy research. Front. Psychiatry.

[31] Goldberg SB (2020). Machine learning and natural language processing in psychotherapy research: Alliance as example use case. J. Couns. Psychol..

[32] Imel ZE, Caperton DD, Tanana M, Atkins DC (2017). Technology-enhanced human interaction in psychotherapy. J. Couns. Psychol..

[33] Goldberg SB (2021). Can a computer detect interpersonal skills? Using machine learning to scale up the Facilitative Interpersonal Skills task. Psychother. Res..

[34] Zhou Y (2022). Predicting first session working alliances using deep learning algorithms: A proof-of-concept study for personalized psychotherapy. Psychother. Res..

[35] Deits-Lebehn C, Baucom KJW, Crenshaw AO, Smith TW, Baucom BRW (2020). Incorporating physiology into the study of psychotherapy process. J. Counsel. Psychol..

[36] Mikkelsen MB, O’Toole MS (2023). A review of bodily dysfunction in depression: Empirical findings, theoretical perspectives, and implications for treatment. J. Psychother. Integr..

[37] First MB, Gibbon M, Hilsenroth MJ, Segal DL (2004). The structured clinical interview for DSM-IV axis I disorders (SCID-I) and the structured clinical interview for DSM-IV axis II disorders (SCID-II)). Comprehensive Handbook of Psychological Assessment.

[38] Ramos MAF (2008). Análise das Caraterísticas Psicométricas da Versão Portuguesa do Working Alliance Inventory- Short Revised.

[39] Hatcher RL, Gillaspy JA (2006). Development and validation of a revised short version of the working alliance inventory. Psychother. Res..

[40] Coutinho J (2017). Psychophysiological reactivity in couples during a marital interaction task. Appl. Psychophysiol. Biofeedback.

[41] Grady NW (2016). Knowledge discovery in data in construction projects. Arch. Civil Eng..

[42] Chapman, P. *et al.* Crisp-Dm 1.0. *CRISP-DM Consortium* 76 (2000).

[43] Huber S, Wiemer H, Schneider D, Ihlenfeldt S (2019). DMME: Data mining methodology for engineering applications—A holistic extension to the CRISP-DM model. Procedia CIRP.

[44] Saidi R, Bouaguel W, Essoussi N, Hassien A (2019). Hybrid feature selection method based on the genetic algorithm and pearson correlation coefficient. Machine Learning Paradigms: Theory and Application.

[45] Michalewicz Z, Shcmidt M, Michalewicz M, Chiriac C (2006). Adaptive Business Intelligence.

[46] Delen D (2020). Prescriptive Analytics The Final Frontier for Evidence-Based Management and Optimal Decision.

[47] Stulp F, Sigaud O (2015). Many regression algorithms, one unified model: A review. Neural Netw..

[48] Parvandeh S, Yeh H-W, Paulus MP, McKinney BA (2020). Consensus features nested cross-validation. Bioinformatics.

[49] Koch P, Wujek B, Golovidov O, Gardner S (2017). Automated Hyperparameter Tuning for Effective Machine Learning.

[50] Krstajic D, Buturovic LJ, Leahy DE, Thomas S (2014). Cross-validation pitfalls when selecting and assessing regression and classification models. J. Cheminform..

[51] Botchkarev A (2019). A new typology design of performance metrics to measure errors in machine learning regression algorithms. Interdiscip. J. Inf. Knowl. Manag..

[52] Quinino RC, Reis EA (1962). Using the coefficient of determination R2 to test the significance of multiple linear regression. Int. J. Stat. Data Sci. Teach..

[53] Mantovani, R. G., Horvath, T., Cerri, R., Vanschoren, J. & de Carvalho, A. C. P. L. F. Hyper-Parameter Tuning of a Decision Tree Induction Algorithm. In *Proceedings of the 2016 5th Brazilian Conference on Intelligent Systems, BRACIS 2016* 37–42 (2017). 10.1109/BRACIS.2016.018.

[54] Huang, Q., Mao, J. & Liu, Y. An improved grid search algorithm of SVR parameters optimization. In *International Conference on Communication Technology Proceedings, ICCT* (2012). 10.1109/ICCT.2012.6511415.

[55] Lee SLA, Kouzani AZ, Hu EJ (2010). Random forest based lung nodule classification aided by clustering. Comput. Med. Imaging Graph..

[56] Rodriguez-Galiano V, Sanchez-Castillo M, Chica-Olmo M, Chica-Rivas M (2015). Machine learning predictive models for mineral prospectivity: An evaluation of neural networks, random forest, regression trees and support vector machines. Ore. Geol. Rev..

[57] Probst P, Wright MN, Boulesteix A (2019). Hyperparameters and tuning strategies for random forest. WIREs Data Min. Knowl. Discov..

[58] Naji A, Abboud SA, Jumaa BA, Abdullah MN (2022). Gait classification using machine learning for foot disseises diagnosis. Tech. Rom. J. Appl. Sci. Technol..

[59] Twumasi C, Twumasi J (2022). Machine learning algorithms for forecasting and backcasting blood demand data with missing values and outliers: A study of Tema General Hospital of Ghana. Int J Forecast.

[60] Kavitha S, Varuna S & Ramya R. A comparative analysis on linear regression and support vector regression. In *Proceedings of 2016 Online International Conference on Green Engineering and Technologies, IC-GET 2016* 1–5 (2017). 10.1109/GET.2016.7916627.

[61] Kumari K, Yadav S (2018). Linear regression analysis study. J. Pract. Cardiovasc. Sci..

[62] Quandt RE (1958). The estimation of the parameters of a linear regression system obeying two separate regimes. J. Am. Stat. Assoc..

[63] Menapace A, Zanfei A, Righetti M (2021). Tuning ANN hyperparameters for forecasting drinking water demand. Appl. Sci..

[64] Tinuke molewa O, Taye Oladele A, Adekanmi Adeyinka A, Roseline Oluwaseun O (2019). Prediction of student’s academic performance using k-means clustering and multiple linear regressions. J. Eng. Appl. Sci..

[65] Valenza G, Citi L, Saul JP, Barbieri R (2018). Measures of sympathetic and parasympathetic autonomic outflow from heartbeat dynamics. J. Appl. Physiol..

[66] Moise N (2018). Patient preferences for personalized (N-of-1) trials: A conjoint analysis. J. Clin. Epidemiol..

[67] Uijtdehaage SHJ, Thayer JF (2000). Accentuated antagonism in the control of human heart rate. Clin. Auton. Res..

[68] Porges SW (2001). The polyvagal theory: Phylogenetic substrates of a social nervous system. Int. J. Psychophysiol..

[69] Porges SW (2007). The polyvagal perspective. Biol. Psychol..

[70] Porges SW (2003). The Polyvagal Theory: Phylogenetic contributions to social behavior. Physiol. Behav..

[71] Butler EA, Wilhelm FH, Gross JJ (2006). Respiratory sinus arrhythmia, emotion, and emotion regulation during social interaction. Psychophysiology.

[72] Kiema H, Rantanen A, Laukka S, Siipo A, Soini H (2014). The connection between skilled counseling and client’s heart rate variability. Procedia Soc. Behav. Sci..

[73] Mather M, Thayer JF (2018). How heart rate variability affects emotion regulation brain networks. Curr. Opin. Behav. Sci..

[74] Quintana DS, Heathers JAJ (2014). Considerations in the assessment of heart rate variability in biobehavioral research. Front. Psychol..

[75] Goodyke MP, Hershberger PE, Bronas UG, Dunn SL (2022). Perceived social support and heart rate variability: An integrative review. West J. Nurs. Res..

[76] Flückiger C, Del Re AC, Wampold BE, Horvath AO (2018). The alliance in adult psychotherapy: A meta-analytic synthesis. Psychotherapy.

[77] Atzil-Slonim D (2015). Therapeutic bond judgments: Congruence and incongruence. J. Consult Clin. Psychol..

[78] Patterson CL, Uhlin B, Anderson T (2008). Clients’ pretreatment counseling expectations as predictors of the working alliance. J. Couns Psychol..

[79] Kleinbub JR (2017). State of the art of interpersonal physiology in psychotherapy: A systematic review. Front. Psychol..

[80] Ng MY, Weisz JR (2016). Annual Research Review: Building a science of personalized intervention for youth mental health. J. Child Psychol. Psychiatry.

[81] Akerkar, R. Symposium on AI, Data and Digitalization. Western Norway Research Institute/Vestlandsforsking (2023).

[82] Rollmann I, Gebhardt N, Stahl-Toyota S, Simon J, Sutclie M, Friederich H-C, Nikendei C (2023). Systematic review of machine learning utilization within outpatient psychodynamic psychotherapy research. Front. Psychiatry.

[83] Schröder-Pfeifer, P. *Machine Learing Applications in Psychotherapy Research*. Doctoral thesis submitted Faculty of Behavioral and Cultural Studies Heidelberg University, (2020).

[84] King MW, Resick PA (2014). Data mining in psychological treatment research: A primer on classification and regression trees. J. Consult Clin. Psychol..

[85] Landin-Romero R, Moreno-Alcazar A, Pagani M, Amann BL (2018). How does eye movement desensitization and reprocessing therapy work? A systematic review on suggested mechanisms of action. Front. Psychol..

